# *Cis*‐acting DNA elements flanking the variable major protein expression site of *Borrelia hermsii* are required for murine persistence

**DOI:** 10.1002/mbo3.569

**Published:** 2017-12-17

**Authors:** Allison E. James, Artem S. Rogovskyy, Michael A. Crowley, Troy Bankhead

**Affiliations:** ^1^ Paul G. Allen School for Global Animal Health Washington State University Pullman WA USA; ^2^ Department of Veterinary Microbiology and Pathology Washington State University Pullman WA USA; ^3^ Present address: Department of Veterinary Pathobiology College of Veterinary Medicine & Biomedical Sciences Texas A&M University College Station TX USA

**Keywords:** antigenic variation, *Borrelia*, genetic recombination, relapsing fever, spirochete

## Abstract

In *Borrelia hermsii*, antigenic variation occurs as a result of a nonreciprocal gene conversion event that places one of ~60 silent variable major protein genes downstream of a single, transcriptionally active promoter. The upstream homology sequence (UHS) and downstream homology sequence (DHS) are two putative *cis*‐acting DNA elements that have been predicted to serve as crossover points for homologous recombination. In this report, a targeted deletion/*in cis* complementation technique was used to directly evaluate the role for these elements in antigenic switching. The results demonstrate that deletion of the expression site results in an inability of the pathogen to relapse in immunocompetent mice, and that the utilized technique was successful in producing complemented mutants that are capable of antigenic switching. Additional complemented clones with mutations in the UHS and DHS of the expressed locus were then generated and evaluated for their ability to relapse in immunocompetent mice. Mutation of the UHS and inverted repeat sequence within the DHS rendered these mutants incapable of relapsing. Overall, the results establish the requirement of the inverted repeat of the DHS for antigenic switching, and support the importance of the UHS for *B. hermsii* persistence in the mammalian host.

## 1 INTRODUCTION

Tick‐borne relapsing fever is caused by a number of spirochete species in the genus *Borrelia* (Barbour, 1990; Stoenner, Dodd, & Larsen, 1982). Human infection with relapsing fever spirochetes results in a series of febrile episodes that are interrupted by periods of apparent wellness. The waxing and waning fever that is characteristic of relapsing fever is a direct result of spirochetal antigenic variation and sequential immune evasion. Febrile episodes correspond to outgrowths of spirochetal populations that possess a predominant serotype. Bacterial density during these episodes can be >10^7^ *Borrelia* per ml of blood, while afebrile periods are marked by serotype‐specific immune responses that greatly reduce or eliminate the bacterial load in blood (Dworkin, Schwan, & Anderson, 2002; Raffel, Battisti, Fischer, & Schwan, 2014). The serotypes of relapsing fever *Borrelia* are characterized by antigenically distinct immunodominant outer surface lipoproteins (Barbour, Tessier, & Stoenner, 1982; Barstad, Coligan, Raum, & Barbour, 1985). Thus, newly “switched” spirochetes are not yet recognized by the host's immune system, and serve as progenitors for the next febrile relapse. The lagging host humoral response will clear the new serotype in time, but the cyclic proliferation of immune escape variants and subsequent immune clearance of these populations will be repeated due to the process of antigenic variation (Barbour & Restrepo, 2000). Not only is antigenic switching linked to the clinical course of disease, it serves to prolong the time in which the bacteria are found in blood. This persistence is an essential feature of the lifecycle of these pathogens, as it increases the chances for tick acquisition (Barbour & Restrepo, 2000; Lopez, Mccoy, Krajacich, & Schwan, 2011). Moreover, it has been demonstrated that without an antigenic variation system, relapsing fever *Borrelia* are unable to persist in mammalian hosts, and therefore, unable to cause febrile relapses (Raffel et al., 2014).

The mechanisms underlying antigenic variation are best characterized in the relapsing fever species *Borrelia hermsii*. In this species, two nonhomologous, immunodominant lipoproteins interchangeably and sequentially confer serotype specificity: the variable large proteins (Vlp) and variable small proteins (Vsp) (Hinnebusch, Barbour, Restrepo, & Schwan, 1998; Restrepo, Kitten, Carter, Infante, & Barbour, 1992). Together, the lipoproteins are known as variable major proteins (Vmp), and the *B. hermsii* genome harbors at least 59 silent *vmp* gene copies (Dai et al., 2006). Only one locus is transcriptionally active during infection of the mammalian host, and this expression site (termed *vmp*_*Ex*_ for the purposes of this study) is located near the telomere end of an approximately 27.8 kb linear plasmid named lpE27 (formerly lp28‐1) (Barbour, 2016; Barbour, Burman, Carter, Kitten, & Bergstrom, 1991; Kitten & Barbour, 1990; Plasterk, Simon, & Barbour, 1985). Antigenic variation occurs through a nonreciprocal gene conversion event that duplicates one of the silent, promoterless *vmp*s downstream of the active promoter at *vmp*_*Ex*_ (Barbour, Burman, et al., 1991; Plasterk et al., 1985). Two *cis*‐acting DNA elements have been implicated as crossover points for homologous recombination, and are found flanking both *vmp*_*Ex*_ and silent *vmp* loci (Barbour, Burman, et al., 1991; Dai et al., 2006; Kitten & Barbour, 1990). The first site, the upstream homology sequence (UHS), is 61–62 bp in length, and is partially intragenic extending 7 bp upstream of the transcriptional start site and 26 bp into the Vmp coding region (Barbour, Burman, et al., 1991; Dai et al., 2006). The second element, the 214 bp downstream homology sequence (DHS), is entirely extragenic and lies a variable distance away from the Vmp stop codon (Dai et al., 2006; Kitten & Barbour, 1990; Restrepo et al., 1992). Within the DHS, a 31‐bp inverted repeat sequence is found between positions 47 and 77 (Dai et al., 2006). Inverted repeat (IR) sequences form putative secondary stem loop structures in DNA, and these structures are known to be highly recombinogenic (Barbour, Dai, Restrepo, Stoenner, & Frank, 2006).

Dai et al. (2006) proposed the following mechanism for antigenic switching in *B. hermsii* based on sequencing analysis: recombination is initiated in the distal portion of the DHS, and the replication fork for repair extends upstream using a silent *vmp* as a template until it reaches the UHS, where replication is terminated. This 3′–5′ mechanism is supported by two key findings. First, a chimeric *vmp*_*Ex*_ site was found in a relapse variant where the upstream portion remained the same as the starting serotype and the downstream portion represented a “switched” *vmp*, indicative of premature replication fork termination (Dai et al., 2006). Second, the initiation of recombination in the DHS is supported by the finding that the *vmp*_*Ex*_ is oriented such that the DHS lies near the telomere of lpE27, a structure that is known to be inherently recombinogenic (Chaconas, Stewart, Tilly, Bono, & Rosa, 2001). A role for the UHS and DHS in the rate of genetic recombination has also been verified. Greater sequence similarity in the UHS and a shorter distance from the stop codon of the *vmp* to its downstream DHS predict a higher recombination rate of the archived *vmp* into the expression site (Barbour et al., 2006).

The objective for the study presented herein was to verify the importance of these *cis*‐acting DNA elements for gene conversion. Thus, we hypothesized that the UHS and DHS are required for the recombination event that leads to antigenic variation. The underlying principle behind our method is that without a functional antigenic variation system, *B. hermsii* is unable to persist in the murine host. Indeed, Raffel et al. (2014) demonstrated that when the *vmp*_*Ex*_ promoter was disrupted, *B. hermsii* was no longer capable of relapsing in the murine host. To start, we employed a direct, mutational approach whereby the *vmp*_*Ex*_ locus was deleted from lpE27 in wild‐type *B. hermsii* using a targeted deletion technique. As expected, the *vmp*_*Ex*_ deletion mutant was not capable of persistence in an immunocompetent murine host, but did persist in severe combined immunodeficient (SCID) mice, indicating that immune clearance in the former is a direct result of the absence of antigenic switching. Next, a novel targeted deletion/*in cis* complementation technique was used to directly evaluate the role of the putative *cis*‐acting sites in antigenic switching. The results from this work strongly support the requirement of the DHS‐IR for antigenic switching in *B. hermsii*, and suggest that the UHS is important for both proper expression and gene conversion of the *vmp*_*Ex*_ locus. Importantly, the findings corroborate, through mutational analysis, the mechanism for *vmp*_*Ex*_ recombination proposed by previous investigators (Dai et al., 2006).

## 2 MATERIALS AND METHODS

### 2.1 Ethics statement

The experimental procedures involving strains of inbred mice were carried out in accordance with the American Association for Accreditation of Laboratory Animal Care (AAALAC) protocol and the institutional guidelines set by the Office of Campus Veterinarian at Washington State University (Animal Welfare Assurance A3485‐01 and USDA registration number 91‐R‐002). Washington State University AAALAC and institutional guidelines are in compliance with the U.S. Public Health Service Policy on Humane Care and Use of Laboratory Animals. Inbred mice were maintained at Washington State University (Pullman, WA, USA) in an AAALAC‐accredited animal facility. The Washington State University Institutional Animal Care and Use Committee reviewed and approved the animal protocols associated with the current studies.

### 2.2 Murine infection

Male, C.B‐17/IcrHsd‐Prkdcscid (SCID) or C3H/HeNHsd (C3H) mice 4–6 weeks of age were purchased from Harlan (Indianapolis, IN). While the SCID mice are from a different background than the wild‐type mice, various strains of SCID mice infected with *B. hermsii* demonstrate similar, persistently high levels of spirochetemia following inoculation (Alugupalli et al., 2003; James, Rogovskyy, Crowley, & Bankhead, 2016; Raffel et al., 2014). The animals were subcutaneously inoculated with a *B. hermsii* clone with 1 × 10^6^ total spirochetes per mouse in 100 μl. All clones, including wild type, were obtained by serial dilution and plating. After plating, they were passaged no more than two times in vitro from frozen glycerol stocks prior to murine inoculation. To confirm infection, 15 μl of blood was drawn from mice via the saphenous vein each day over the course of 10 days. A total of 5 μl of blood was immediately diluted 1:3 with BSK‐II medium (Barbour, 1984), and then 10 μl of diluted blood was examined under a dark field microscope. The remaining 10 μl of whole blood was cultured in 1 ml of BSK‐II containing *Borrelia* antibiotic cocktail (0.02 mg/ml phosphomycin, 0.05 mg/ml rifampicin, and 2.5 mg/ml amphotericin B). Blood cultures were incubated at 35°C under 1.5% CO_2_ for 3–4 weeks. The blood cultures were periodically examined via dark‐field microscopy for the presence of viable *Borrelia* cells. A mouse whose blood sample(s) showed viable spirochetes via culture and/or microscopy was considered infected.

### 2.3 Bacterial strains, culture conditions, and DNA extraction

The isolation and characterization of *B. hermsii* DAH has been described previously (Porcella et al., 2005; Schwan, Schrumpf, Hinnebusch, Anderson, & Konkel, 1996), and was acquired as a gift from George Chaconas, who obtained it from Tom Schwan. The DAH strain is nearly identical to isolate HS1, with the latter being the subject of many previous studies on antigenic variation in *B. hermsii* (Barbour, 2016; Raffel et al., 2014). All wild‐type and mutant clones of *B. hermsii* were cultivated at 35°C under 1.5% CO_2_ in modified Barbour–Stoenner–Kelly medium (BSK‐II) supplemented with 12% rabbit serum (Accurate Chemical and Scientific Corp., Westbury, NY). Cell densities and growth phase were monitored under dark‐field microscopy and enumerated using a Petroff‐Hausser counting chamber. DNA from *B. hermsii* used in Southern blotting, field inversion gels, and PCR analysis was extracted from in vitro grown cultures using a plasmid midi kit (Qiagen, Valencia, CA).

All plasmids generated herein were propagated in EC19 *Escherichia coli* cells (Hove, Haldorson, Magunda, & Bankhead, 2014). To obtain clonal colonies after transformation into *E. coli*, plates were incubated at 35°C overnight. All cultures in liquid broth were incubated with shaking overnight at 30°C. Plasmids for further cloning or sequence verification were extracted using a plasmid midi kit (Qiagen). Plasmids for transformation into *B. hermsii* were extracted using a midi kit (Qiagen), and further ethanol precipitated to concentrate the DNA.

### 2.4 Targeted deletion plasmid construction

A plasmid vector (pAE12) was constructed for the deletion of *vmp*_*Ex*_ using a targeted deletion technique that has been described previously in *Borrelia burgdorferi* (Bankhead & Chaconas, 2007; Beaurepaire & Chaconas, 2005; Chaconas et al., 2001). To construct pAE12, the *B. burgdorferi* replicated telomere (*rtel*) of pYT1 (Tourand, Kobryn, & Chaconas, 2003) was first excised via BamHI digestion. The pYT1 minus *rtel* construct was then subjected to inverse PCR amplification with phosphorylated primers P237 and P259 (primers listed in Table S1). Each of these primers possessed half of the *B. hermsii* lpE27 replicated telomere as 5′ tails (Kitten & Barbour, 1990; Tourand et al., 2003). The resulting amplicon was self‐ligated, producing pAE2. After linearization of pAE2 with PstI, the *rtel* was verified following treatment with purified *B. hermsii* ResT (Kobryn & Chaconas, 2002). Next, a *B. hermsii flgB* promoter‐*aphI* gene conferring kanamycin resistance (*kan* ^*R*^) was amplified with P245 and P246 using template plasmids described elsewhere (James et al., 2016). These primers possessed NgoMIV and NheI 5′ tails, which allowed for replacement of the *B. burgdorferi flgB* promoter‐driven *kan* ^*R*^ with a *B. hermsii flgB*p/*kan* ^*R*^, producing plasmid pAE4. To remove extraneous DNA sequence, the entire *B. hermsii flgBp/kan* ^*R*^ segment on pAE4 was amplified with P422 and P423, as well as the *E. coli* origin of replication with P424 and P425. The two products were ligated together using PmeI and ScaI sites, producing pAE11. P423 and P424 also possessed XhoI and AscI sites, respectively, which allowed for the cloning of the 1,005 bp *B. hermsii* lpE27 target sequence that was amplified with P395 and P396. According to the annotated *B. hermsii* DAH lpE27 sequence in the NCBI GenBank database (accession number CP000273 Dai et al., 2006, http://www.ncbi.nlm.nih.gov/), this target sequence lies between base pairs 1,382 and 2,386. The final targeted deletion plasmid, pAE12, was verified via sequencing.

### 2.5 Purification of *B. hermsii* telomere resolvase

*Borrelia hermsii* telomere resolvase (ResT) was obtained by cloning the *resT* amplicon produced with primers P274 and P275 into pET15b (Novagen‐Merck Millipore, Darmstadt, Germany) using BamHI and NdeI sites, respectively. The pET15b/*resT* construct was transformed into Rosetta 2(DE3)pLysS *E. coli* competent cells (Novagen), and ResT was purified using previously published methods (Kobryn & Chaconas, 2002).

### 2.6 Complementation and UHS/DHS mutant plasmid construction

The plasmid pAE160 was generated for *in cis* restoration of *vmp*_*Ex*_ on native plasmid lpE27 using the targeted deletion technique. Utilizing the XhoI and AscI restriction enzyme sites on pAE12, the target sequence was replaced by cloning a PCR amplified downstream portion of lpE27 with primers P626 and P627, producing pAE16. This 986 base pair target sequence lies between base pairs 10,339 and 11,324 on the GenBank annotated *B. hermsii* DAH lpE27 plasmid (accession number CP000273). Next, the variable membrane protein expression site, *vmp*_*Ex*_, was amplified with P541 and P542, and cloned into pAE16 using existing NheI and FspI sites, respectively. The resultant plasmid, pAE160, was then transformed into wild‐type *B. hermsii*, producing the complemented mutant.

To generate plasmids that have mutated UHS and DHS, *vmp*_*Ex*_ sites with different sequence alterations were commercially synthesized (GenScript, Piscataway, NJ) and cloned into pAE160 using NheI and FspI sites. By replacing the native *vmp*_*Ex*_ with the mutated versions, plasmids pAE160UHS and pAE160DHS were generated. All plasmids were sequenced and verified prior to transformation into wild‐type *B. hermsii*.

### 2.7 *Borrelia hermsii* transformation and mutant screening

*Borrelia hermsii* DAH electrocompetent cells were prepared and electroporated according to methods described previously (Fine, Earnhart, & Marconi, 2011; Samuels, 1995). Following electroporation, the cells were treated as outlined in James et al. (2016) to obtain clonal populations. Briefly, electroporated cells were immediately placed in 5 ml of 35°C BSK‐II, where they were allowed to recover overnight. The 5 ml recovery culture was then added to 45 ml of fresh, preincubated BSK‐II under kanamycin selection (200 μg/ml). Once viable spirochetes were observed in this polyclonal culture, clonal isolation of transformants was achieved through serial dilution in fresh BSK‐II with kanamycin, and plating in 96‐well culture plates (Sarstedt, Newton, NC).

Clonal populations of mutants were initially identified by a change of media color from red to yellow. Positive wells were PCR screened for the presence of *kan* ^*R*^, and correct clones were subcultured in 50 ml fresh BSK‐II with kanamycin for DNA extraction. DNA from the mutants was subjected to PCR for the expected lpE27 integration site. Primers P233 and P537 were used to amplify and sequence the integration site of *Bh*∆*vmp*_*Ex*_; primers P854 and P855 were used for *Bh*::Comp, *Bh*::UHS_AS_, and *Bh*::DHS_ΔIR_. All mutant DNA was also PCR amplified with P308 and P309 to verify the absence of *vmp*_*Ex*_ in *Bh*∆*vmp*_*Ex*_, and to sequence *vmp*_*Ex*_ in the other mutants.

### 2.8 Southern blotting

Total plasmid DNA (200 ng) from wild‐type *B. hermsii* and all mutant clones was electrophoresed on a 1.7% agarose gel at 80 volts for 22.5 hr, and transferred to a nylon membrane (GE Healthcare, Buckinghamshire, UK). Digoxigenin‐labeled probes (*kan* ^*R*^ primers 5′‐CATATGAGCCATATTCAACGGGAAACG‐3′ and 5′‐AAAGCCGTTTCTGTAATGAAGGAG‐3′; lpE27 primers 5′‐CTTCCTTAAATTTGTTCGGCCCCGA‐3′ and 5′‐CGACATGGATGCCCAAGCAAGTCT‐3′) were used for DNA hybridization according the reagent manufacturer's instructions (Roche, Indianapolis, IN).

### 2.9 Antibody generation

The gene of the current Vmp expressed in our stock culture of *B. hermsii* DAH, *vlpA7*, was amplified with P558 and P559, possessing NdeI and BamHI 5′ tails, respectively. The 1,016 base pair product was cloned into pET15b (Novagen‐Merck Millipore), and recombinant protein was obtained by Ni‐NTA agarose column protein purification (Qiagen). Proteins were concentrated with Pierce Protein Concentrators 9K MWCO and quantified with a Micro BCA Protein Assay kit (Thermo Scientific, Marietta, OH). Recombinant VlpA7 (58.5 μg) was mixed with TiterMax Gold Adjuvant (Sigma Aldrich, St. Louis, MS) following the manufacturer's instructions, and injected subcutaneously into three C3H/HeNHsd mice (Harlan, Indianapolis, IN). Thirty‐four days post inoculation, the mice were exsanguinated and humanely euthanized. Collected blood was centrifuged at 6,000*g* for 10 min, serum was pooled, and stored at −80°C until use in immunoblotting.

### 2.10 Western blotting

Wild‐type or mutant *B. hermsii* were grown to late‐log phase, counted, and centrifuged (6,000*g* for 15 min) to achieve duplicate pellets of 1 × 10^9^ cells. Pellets were resuspended in 450 μl PBS + 5 mmol/L MgCl_2_. Eight units of proteinase K were added to one sample and incubated at room temperature for 40 min. The second aliquot of cells served as an untreated control. Ten microliter of phenylmethanesulfonylfluoride (0.2 mol/L) was added to inhibit further proteinase K activity, and the cells were washed in 500 μl of PBS‐MgCl_2_ two times. Cells were resuspended in 950 μl PBS‐MgCl_2_ and 500 μl of sodium dodecyl sulfate‐polyacrylamide gel electrophoresis (SDS‐PAGE) sample buffer (Rogovskyy & Bankhead, 2013). Lysates were stored at −20°C until use.

Ten microliter of each lysate (~1 × 10^7^ cells) was heated at 100°C for 5 min prior to loading on a 15% acrylamide gel. Samples were electrophoresed in a Tris‐glycine buffer containing 0.01% SDS. Resolved proteins were transferred to a 0.45‐μm pore nitrocellulose membrane (Bio‐Rad, Hercules, CA). The membranes were blocked in 5% nonfat dry milk in TBS overnight at 4°C, then incubated with either 1:750 diluted rabbit anti‐*B. burgdorferi* FlaB antibody (Rockland Immunochemicals, Gilbertsville, PA) or 1:1,000 anti‐VlpA7 antibody at room temperature for 1 hr. The membranes were washed three times with TBST, then incubated at room temperature for 1 hr with a 1:5,000 dilution of either donkey anti‐mouse or anti‐rabbit horseradish peroxidase‐conjugated secondary antibody (Jackson ImmunoResearch Laboratories, West Grove, PA). After washing two times with TBST and once with TBS, the blots were developed using an enhanced chemiluminescent substrate (Bio‐Rad).

### 2.11 Field inversion gel electrophoresis

Plasmid DNA (500 ng) was electrophoresed on a 0.7% SeaKem LE Agarose gel (Lonza, Basel, Switzerland) with 0.5× TBE buffer. DNA was initially electrophoresed without inversion at 100 V for 15 min, followed by 24 hr of inversion with recirculation of buffer at 4°C. The reverse‐pulse electrical field was supplied by a PPI‐200 Programmable Power Inverter (MJ Research, Watertown, MA) on Program 3.

### 2.12 In vitro growth assays

Wild‐type *B. hermsii* and all mutant clones were grown to late‐log phase and subcultured in triplicate to a cell density of 1 × 10^5^ spirochetes per ml. Spirochetes were enumerated at 24‐hr intervals and expressed as mean densities with standard deviation. Cell densities and growth phase were monitored by visualization under dark‐field microscopy and enumerated using a Petroff‐Hausser counting chamber.

### 2.13 Sequencing of *vmp*_*Ex*_

The *vmp*_*Ex*_ locus of spirochetes recovered from exponential‐phase murine blood cultures were sequenced to assess antigenic switching. Blood cultures were diluted 1:5 in PBS, and subjected to standard PCR with primers P308 and P309 (Table S1). The resultant *vmp*_*Ex*_ amplicon was cleaned using the QIAquick PCR Purification Kit (Qiagen), and submitted for sequencing using the “Power Read” option for difficult templates (Eurofins Operon, eurofinsgenomic.com). For inconclusive sequencing results, the *vmp*_*Ex*_ amplicon was cloned into pJET1.2/blunt (Thermo Fisher Scientific, Waltham, MA), transformed into DH5‐α *Escherichia coli*, and plated to obtain individual colonies. Five individual colony‐forming units from each blood culture were inoculated into Luria broth media and incubated overnight at 37°C. The pJET 1.2/*vmp*_*Ex*_ amplicon constructs were extracted with a QIAprep Mini kit (Qiagen), and submitted for sequencing as described above.

### 2.14 Statistical analyses

All analyses were performed using the R statistical platform (version 3.4.0, R Core Team, 2017). Linear regression and analysis of covariance was applied for statistical comparison of growth curves. Analysis of variance (ANOVA) was used to compare the mean maximum cell density of each strain to one another. Fisher's exact test was used in cases where data could be applied to contingency tables (murine infection data). The *p*s < .05 were considered statistically significant.

## 3 RESULTS

### 3.1 Targeted deletion of the *B. hermsii vmp*_*Ex*_ locus

A recent study by Raffel et al. (2014) demonstrated that *vmp*_*Ex*_ can be disrupted through the replacement of the promoter and 5′ region of *vmp*_*Ex*_ with an antibiotic resistance cassette. In order to verify the importance of the UHS and DHS in antigenic switching for this study, a construct with an intact promoter region was required. Thus, our methods differed from the previously published study in that we employed a targeted deletion technique, and later, a similar *in cis* complementation technique to manipulate *vmp*_*Ex*_. To validate our methods, we first genetically deleted the *vmp*_*Ex*_ locus. Targeted deletion has been previously used in *B. burgdorferi*, the causative agent of Lyme disease, but has never been applied to relapsing fever *Borrelia* spp. (Bankhead & Chaconas, 2007; Chaconas et al., 2001).

**Figure 1:**
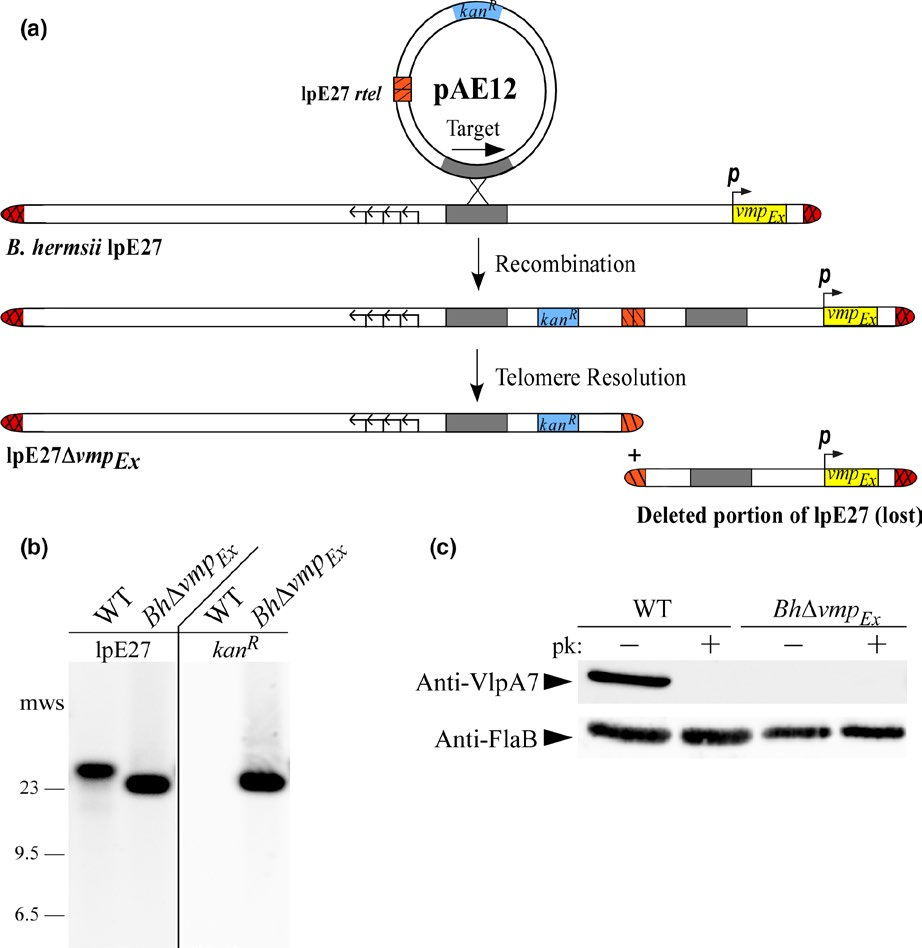
A *Borrelia hermsii* mutant lacking *vmp*_*Ex*_ is generated through targeted deletion. (a) Targeted deletion of *vmp*_*Ex*_ on *B. hermsii* lpE27 is depicted. Following transformation of the targeted deletion construct, pAE12, into wild‐type *B. hermsii*, the plasmid is integrated into lpE27 via homologous recombination at the target site (gray). The introduced replicated telomere (*rtel*, orange stripes) is resolved by endogenous ResT, resulting in a deletion of all downstream sequence, including the entire *vmp*_*Ex*_ promoter (*p*, arrow) and locus. The resulting lpE27 plasmid of *Bh*∆*vmp*_*Ex*_ is truncated and possesses *kan* ^*R*^. Arrows depict the location of genes required for autonomous replication of lpE27. (b) DNA hybridization with an lpE27 specific probe and a *kan* ^*R*^ probe to wild‐type (WT) *B. hermsii* and *Bh*∆*vmp*_*Ex*_ plasmid DNA. Selected molecular weight standards (mws) are shown on left. (c) Surface proteolysis and immunoblot of WT *B. hermsii* and *Bh*Δ*vmp*_*Ex*_ lysates with Anti‐VmpE antibodies. Both clones were proteinase K (pk) treated (+) and untreated (−) to detect surface localization of variable major proteins (Vmp). Anti‐FlaB immunoblots served as a loading control

To utilize targeted deletion for the generation of a *vmp*_*Ex*_^‐^ *B. hermsii* clone (*Bh*∆*vmp*_*Ex*_), the deletion plasmid pAE12 was produced. pAE12 was developed by cloning a target sequence identical to a 1,005 bp region on lpE27 immediately downstream of the genes for autonomous replication into a plasmid construct containing a replicated telomere and an *aphI* gene conferring kanamycin resistance (*kan* ^*R*^; Figure 1a). Replicated telomeres (*rtel*s) are naturally produced during linear plasmid replication in *Borrelia* spp., and DNA cleavage and rejoining of the *rtel* by a telomere resolvase (ResT) results in completion of the linear plasmid duplication event (Kobryn & Chaconas, 2001, 2002). Following transformation of pAE12 into electrocompetent wild‐type *B. hermsii*, the plasmid recombined and integrated into lpE27. Telomere resolution by the enzymatic activity of endogenous ResT on the introduced *rtel* subsequently deleted any downstream sequence, including *vmp*_*Ex*_. In total, lp28‐1 was truncated by approximately 8 kb after targeted deletion. *Borrelia hermsii* mutant clones with a deleted *vmp*_*Ex*_ locus were initially PCR screened for the presence of *kan* ^*R*^ and the absence of *vmp*_*Ex*_ (data not shown). The expected integration site of pAE12 into lpE27 was then PCR amplified and verified by DNA sequencing. Southern blotting with an lpE27‐specific probe further demonstrated that lpE27 was appropriately truncated in *Bh*∆*vmp*_*Ex*_, and that a plasmid of the same size hybridized to a *kan* ^*R*^ probe (Figure 1b). Finally, no expression of Vmp was observed in *Bh*∆*vmp*_*Ex*_ when immunoblotted with anti‐VlpA7 antibody (Figure 1c), which is the serotype of the *B. hermsii* DAH parent strain. Altogether, the results indicate that targeted deletion was effective in deleting the *vmp*_*Ex*_ locus from the *B. hermsii* lpE27 plasmid, resulting in elimination of Vmp expression.

**Figure 2:**
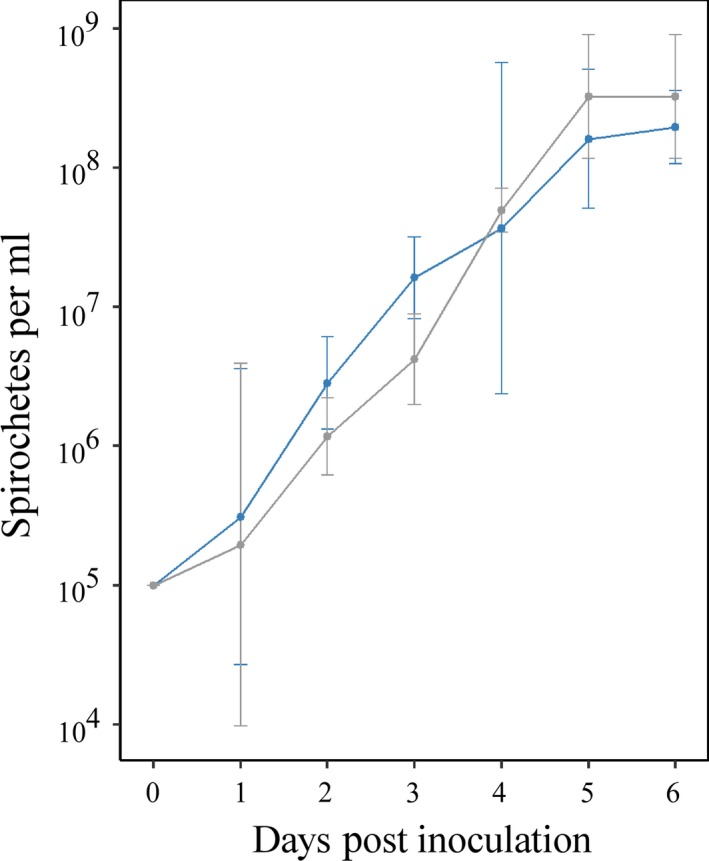
In vitro growth curves of wild‐type *Borrelia hermsii* and *Bh*Δ*vmp*_*Ex*_. Wild‐type *B. hermsii* is shown in gray; *Bh*Δ*vmp*_*Ex*_ is shown in blue. No significant differences in growth rate (*p* = .37) or maximum cell density (*p *= .17), as determined by linear regression analysis of the slopes and analysis of variance, respectively, were detected between *Bh*∆*vmp*_*Ex*_ and wild‐type *B. hermsii* (*p *> 0.05). The mean and 95% confidence intervals (bars) of triplicate measurements at each time point are plotted

To determine whether deletion of *vmp*_*Ex*_ results in altered growth during in vitro cultivation, a growth curve of *Bh*∆*vmp*_*Ex*_ was compared to wild‐type *B. hermsii* (Figure 2). During the 72 hr of exponential‐phase growth (days 1–4 post subculture), the *Bh*∆*vmp*_*Ex*_ doubling time was 10.4 hr (95% CI [8.1, 14.4 hr]), while the doubling time for wild‐type *B. hermsii* was 9.3 hr (95% CI [7.7, 11.7 hr]). Linear regression analysis of the slopes during these 3 days also revealed no significant differences between the growth of the mutant and wild‐type strains (data in Table S2). Mean maximum cell densities for both clones occurred at 6 days post inoculation (wild‐type mean = 3.4 × 10^8^ spirochetes per ml, 95% CI [1.8 × 10^8^, 5.1 × 10^8^]; *Bh*∆*vmp*_*Ex*_ **= 2.0 × 10^8^, 95% CI [1.5 × 10^8^, 2.5 × 10^8^]), and were not significantly different (*p* = .17). Comparison of overall in vitro growth patterns between *Bh*∆*vmp*_*Ex*_ and the wild‐type strain revealed minimal differences, indicating that deletion of *vmp*_*Ex*_ does not result in growth defects.

### 3.2 *Bh*∆*vmp*_*Ex*_ does not establish persistent infection in immunocompetent mice

In principle, *B. hermsii* spirochetes that are unable to express and antigenically switch *vmp*_*Ex*_ cannot persist in an immunocompetent host. In order to establish a proof of principle and to determine whether a *B. hermsii* mutant with a genetically deleted *vmp*_*Ex*_ would be cleared in immunocompetent mice, two groups of five C3H mice were inoculated with 1 × 10^6^ wild‐type *B. hermsii* or *Bh*Δ*vmp*_*Ex*_. While the infectious dose of wild‐type *B. hermsii* can be as low as 1 organism, an inoculum of 10^6^ provided the ability to consistently detect spirochetes in the blood of C3H mice by day 1–2 post inoculation (Dai et al., 2006). Detection of the onset of spirochetemia was longer at increasingly lower doses, and much more variable, a finding confirmed by other investigators (Alugupalli et al., 2003). Blood samples were taken each day from each mouse throughout a 10‐day experimental period. Mice whose blood samples revealed spirochetes either by direct microscopy or culture were considered positive for the day sampled. The results demonstrated that the wild‐type clone was able to persist in all five mice throughout the 10‐day study period, whereas mice infected with *Bh*Δ*vmp*_*Ex*_ remained positive only up to day 5 post inoculation (Table 1). Similarly, two groups of five SCID mice that lack an acquired immune response were inoculated with both clones. *Bh*Δ*vmp*_*Ex*_ mutant spirochetes were capable of persistence in SCID mice throughout the entire study period indicating the mutant does not have any inherent defects in its ability to infect and persist in the absence of an antibody response (Table 1). Consistent with a previously published study (Raffel et al., 2014), the data indicate that the lack of a functional *vmp*_*Ex*_ results in clearance of infection from the blood in immunocompetent hosts, but not in immunodeficient mice. These results further verify the importance of *vmp*_*Ex*_ for evasion of the acquired immune response.

**Table 1:** Infectivity of *Borrelia hermsii* wild type and *Bh*Δ*vmp*_*Ex*_ in C3H and SCID mice

Day post inoculation	C3H mice infected with		SCID mice infected with	
	WT	*Bh*Δ*vmp*_*Ex*_	WT	*Bh*Δ*vmp*_*Ex*_
1	4/5^a^	4/5	5/5	5/5
2	4/5	5/5	5/5	5/5
3	5/5	5/5	5/5	5/5
4	5/5	3/5	5/5	5/5
5	5/5	1/5^b^*	5/5	5/5
6	5/5	0/5**	5/5	5/5
7	5/5	0/5**	5/5	5/5
8	5/5	0/5**	5/5	5/5
9	5/5	0/5**	5/5	5/5
10	5/5	0/5**	5/5	5/5

SCID, severe combined immunodeficient; WT, wild type.

a Value listed corresponds to the number of spirochete positive mice/number tested.

b Statistically significant difference between mutant and wild‐type *B. hermsii* control groups denoted by asterisks as determined by Fisher's exact test (**p < .01, *p < .05).

### 3.3 Generation of an *in cis vmp*_*Ex*_ complement and UHS/DHS mutant clones

#### 3.3.1 Construction of an *in cis vmp*_*Ex*_ complement clone

**Figure 3:**
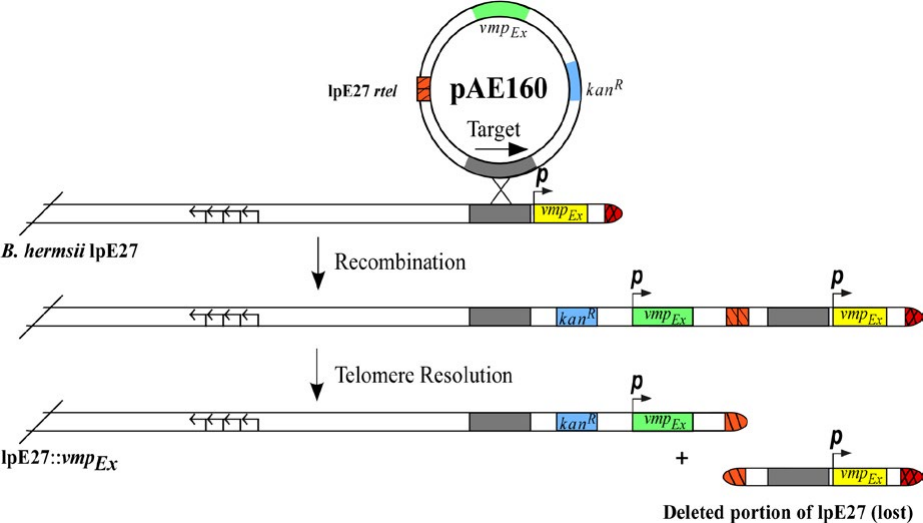
Generation of a functional *in cis vmp*_*Ex*_ complementation mutant. *In cis* complementation of *vmp*_*Ex*_ was achieved by a simultaneous deletion of the native locus and introduction of a cloned *vmp*_*Ex*_. The plasmid pAE160 possesses a target site for homologous recombination that lies just upstream of *vmp*_*Ex*_, a kanamycin resistance locus (*kan* ^*R*^), a replicated telomere (*rtel*, orange stripes), and an introduced *vmp*_*Ex*_ (shown in green). Following homologous recombination at the target sequence, the pAE160 plasmid is integrated into lpE27. Endogenous ResT acts upon the introduced *rtel* and all downstream sequence is deleted, including the native *vmp*_*Ex*_ (yellow). The resultant mutant, *Bh*::Comp, now possesses an lpE27 plasmid with a *vmp*_*Ex*_ and *kan* ^*R*^. The direction and promoters of the *vmp*_*Ex*_ loci are depicted as *p*, with arrows; four arrows depict the location of genes required for autonomous replication of lpE27

In order to test the importance of the UHS and DHS for recombination, it was necessary to ensure that replacement of the native *vmp*_*Ex*_ with an in vitro‐generated locus did not, in itself, disrupt antigenic switching. Thus, an *in cis vmp*_*Ex*_‐complemented mutant (*Bh*::Comp) was created that served to verify that gene conversion was still possible after *vmp*_*Ex*_ restoration with a commercially synthesized gene copy onto the native lpE27 plasmid, and to provide us with the ability to genetically manipulate the UHS and DHS in downstream experiments. Repeated efforts to generate a true complement whereby *vmp*_*EX*_ was restored onto the telomeric end of *Bh*Δ*vmp*_*Ex*_ failed repeatedly to produce transformants. Thus, our complemented strain was generated through a one‐step deletion and *in cis* complementation technique. In doing so, a plasmid to mediate the *in cis* complementation, pAE160, was produced. This plasmid is similar to the targeted deletion plasmid pAE12, except that it possesses the *vmp*_*Ex*_ locus and a 993‐bp target site that lies just upstream of *vmp*_*Ex*_ on lpE27 (Figure 3). Similarly, pAE160 includes *kan* ^*R*^ for selection of transformants and an *rtel*. Following transformation and integration of pAE160 into the lpE27 plasmid of wild‐type *B. hermsii*, endogenous ResT resolves the introduced *rtel*, and the native *vmp*_*Ex*_ is replaced by the introduced, in vitro‐generated *vmp*_*Ex*_ locus. Transformants were initially PCR screened for the presence of *kan* ^*R*^ followed by DNA sequencing of the insertion site.

#### 3.3.2 Construction of UHS/DHS mutants

**Figure 4:**
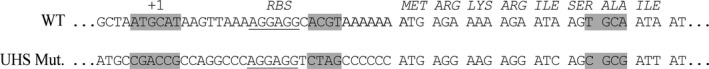
Sequence of *vmp*_*Ex*_ in wild‐type *Borrelia hermsii* and the UHS mutant. The upstream homology sequence (UHS) of the wild‐type (WT) *vmp*_*Ex*_ is shown with the sequence of the mutated UHS (Mut). The transcriptional start site (+1), ribosomal binding site (RBS, underlined), and amino acid codons are depicted at top. Sites of conserved 4‐mer and 6‐mer palindromes are highlighted in gray. The −10 and −35 promoter elements are outside of the putative UHS boundaries and were not altered

Sequencing analysis of infection versus relapse variants has implicated the conserved UHS and DHS elements as crossover points for the recombination event that leads to antigenic variation (Dai et al., 2006). The high degree of sequence similarity in these elements between archived *vmp*s and *vmp*_*Ex*_, along with the DHS‐resident IR sequence, are proposed to be important for initiation and termination of gene conversion. In order to verify the requirement of the UHS and the DHS‐resident IR for antigenic switching, mutants were generated in which the homology of the UHS in the *vmp*_*Ex*_ locus was destroyed, and the IR within the DHS was deleted. To do this, the 1,576 bp *vmp*_*Ex*_ locus was commercially synthesized with incorporated mutations in the UHS and DHS. The mutant *vmp*_*Ex*_ sites were then cloned into pAE160 for transformation into wild‐type *B. hermsii*. For the UHS mutant, all base pairs upstream of the start codon were changed from A to G and T to C, and vice versa, with the exception of the ribosomal binding site and transcriptional start site (the −10 and −35 promoter elements are outside of the UHS region). Homology was destroyed in the intragenic portion of the UHS by targeting only the wobble codon positions (Figure 4). The second mutation eliminated the 31 bp IR that lies within the DHS of *vmp*_*Ex*_. The corresponding constructs, pAE160/UHS, and pAE160/DHS, respectively, were sequenced to verify the inclusion of appropriate UHS/DHS mutations. The pAE160 *vmp*_*Ex*_ mutant plasmids were then transformed into electrocompetent wild‐type *B. hermsii*. A summary of all mutants is listed in Table 2.

**Table 2:** *Borrelia hermsii* mutant clones used in this study

Mutant name	Description
*Bh*∆*vmp*_*Ex*_	*vmp*_*Ex*_ locus is deleted
*Bh*::Comp	*in cis* complement of the native *vmp*_*Ex*_ locus
*Bh*::UHS_AS_	*in cis* complement with the homology of the UHS destroyed in *vmp*_*Ex*_ (AS designation refers to altered sequence)
*Bh*::DHS_ΔIR_	*in cis* complement with the DHS resident IR deleted in *vmp*_*Ex*_

UHS, upstream homology sequence; DHS, downstream homology sequence; IR, inverted repeat.

#### 3.3.3 Verification of *vmp*_*Ex*_ complement and mutant clones

**Figure 5:**
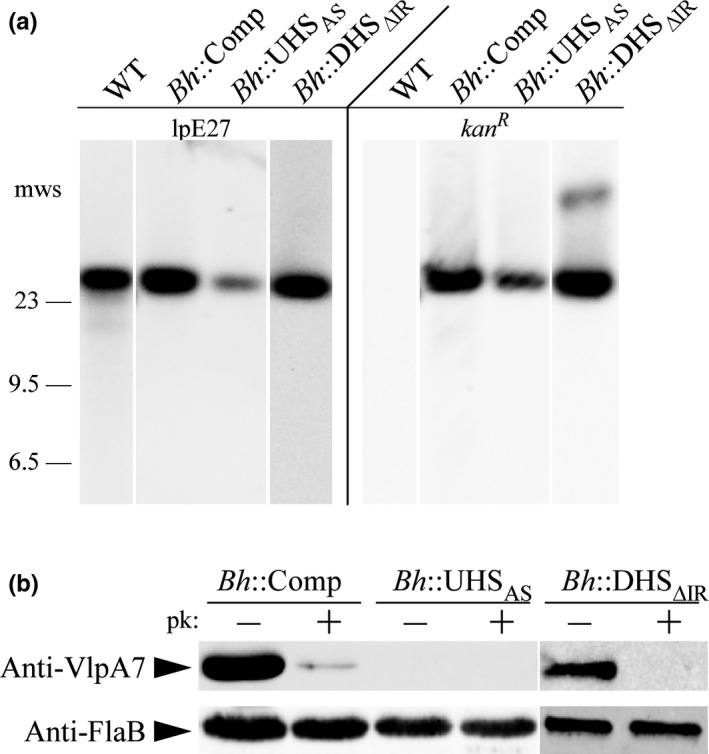
Verification of the *in cis vmp*_*Ex*_ complemented clone and the upstream homology sequence (UHS)/downstream homology sequence (DHS) mutants. (a) DNA hybridization with an lpE27 specific probe and a *kan* ^*R*^ probe to wild‐type (WT) *Borrelia hermsii*,* Bh*::Comp, *Bh*::UHS_AS_, and *Bh*::DHS_ΔIR_ plasmid DNA. Selected molecular weight standards (mws) are shown on left. (b) Surface proteolysis and immunoblot of the *in cis vmp*_*Ex*_ complemented clone and UHS/DHS mutants with Anti‐VlpA7 antibodies. All clones were proteinase K (pk) treated (+) and untreated (−) to detect surface localization of variable major proteins. Anti‐FlaB immunoblots served as a loading control

After transformation with pAE160, pAE160/UHS, or pAE160/DHS, clones were initially screened for the presence of *kan* ^*R*^. The expected integration site of each mutant was PCR amplified and sequenced. Correct clones were then subjected to Southern blotting, surface proteolysis, and immunoblotting. For all complemented mutants, DNA hybridization with *kan* ^*R*^ and lpE27‐specific probes revealed that integration of pAE160 constructs occurred on the *B. hermsii* lpE27 plasmid (Figure 5a). Immunoblotting of *Bh*::Comp and *Bh*::DHS_ΔIR_ lysates with an Anti‐VlpA7 antibody demonstrated binding before, but not after, proteinase K treatment (Figure 5b). Interestingly, the results also showed that the *Bh*::UHS_AS_ does not express Vmp. Together, the results demonstrate that recombination of pAE160 successfully replaced the native *vmp*_*Ex*_ with the introduced *vmp*_*Ex*_ on lpE27, and that Vmp is expressed and surface localized in all but the UHS mutant, similar to wild‐type *B. hermsii*. As a final verification, the introduced *vmp*_*Ex*_ sites in the *B. hermsii* complement and UHS/DHS mutant clones were PCR amplified and sequenced. The *vmp*_*Ex*_ sites in all mutants were found to be correct.

**Figure 6:**
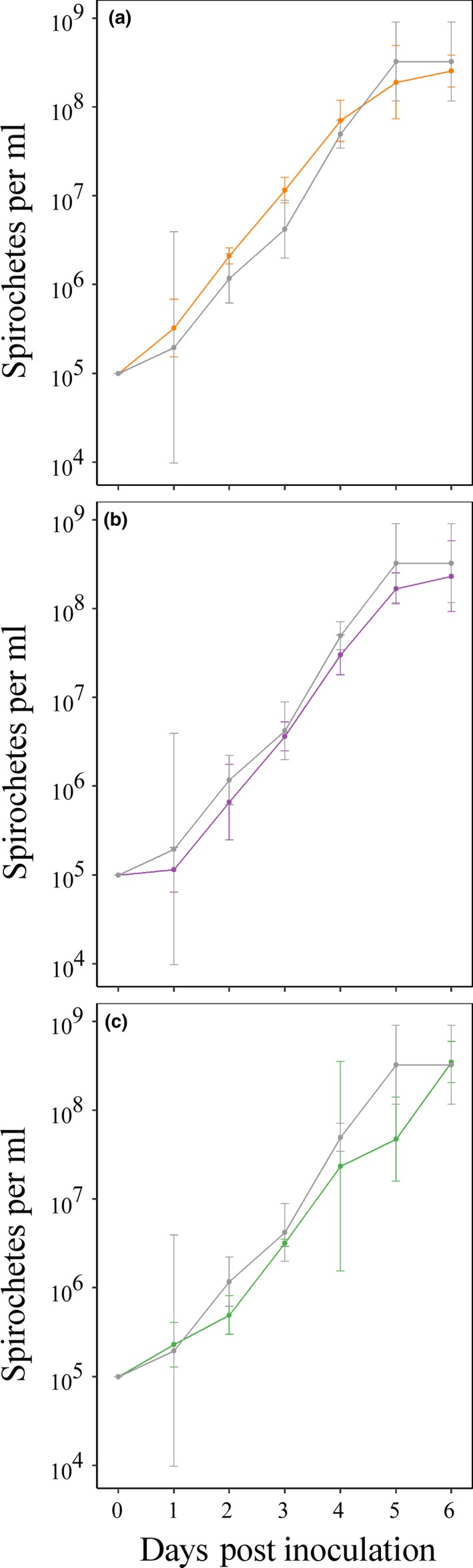
Growth curves of *Bh*::Comp, *Bh*::UHS_AS_, and *Bh*::DHS_ΔIR_ compared to wild‐type *Borrelia hermsii*. The growth curve of wild‐type *B. hermsii* is shown in each panel in gray compared to (a) *Bh*::Comp depicted in orange, (b) *Bh*::UHS_AS_ in purple, (c) and *Bh*::DHS_ΔIR_ in green. No significant differences in growth rate or maximum cell density, as determined by linear regression analysis of the slopes and analysis of variance, respectively, were detected between any of the strains (*p* > .05). The mean and 95% confidence intervals (bars) of triplicate measurements at each time point are plotted

To determine if the mutant clones had replication defects, in vitro growth curves were generated (Figure 6). All mutants were grown to late exponential phase and then subcultured to 1 × 10^5^ spirochetes per ml at the start of the growth curve. During exponential growth (days 1–4 post subculture), the doubling times (and 95% confidence intervals) for *Bh*::Comp, *Bh*::UHS_AS_, and *Bh*::DHS_ΔIR_ were 9.3 (8.8–9.9), 9.0 (8.3–9.8), and 10.5 (8.6–13.6) hr, respectively. Linear regression analysis of the slopes during these 72 hr of exponential growth revealed that none of the mutants had significantly different growth than the wild‐type strain, nor one another (Table S2). Moreover, all mutants reached similar maximum spirochetal densities at day 6 post inoculation, and none of the mutant mean maximum cell densities were significantly different from wild type (data in Table S3). In sum, the data indicate that all *B. hermsii* mutants were successfully generated, and that none of the mutants displayed in vitro growth defects.

### 3.4 A *B. hermsii* mutant with an *in cis*‐complemented *vmp*_*Ex*_ locus persists in immunocompetent mice and undergoes antigenic variation

In order to determine the importance of the UHS and DHS in antigenic switching through *in cis* complementation of mutant *vmp*_*Ex*_ sites, it was essential to first verify that the *Bh*::Comp clone is capable of antigenic variation, and therefore, persistence. To verify this infection phenotype, groups of five C3H or SCID mice were inoculated with 1 × 10^6^ *Bh*::Comp spirochetes. Each mouse was monitored for the presence of spirochetes by blood collection once daily for 10 days. Mice were considered positive when blood samples revealed spirochetes either by direct microscopy or blood culture. As shown in Table 3, *Bh*::Comp exhibited delayed detection in the bloodstream, as they were not found until day 4 post inoculation, while wild‐type *B. hermsii* was detectable by day 1. This delay resulted in a statistically significant difference between wild‐type and *Bh*::Comp in the number of positive mice on day 3 post inoculation. However, by day 6 post inoculation, *Bh*::Comp was detected in the bloodstream of all mice throughout the remainder of the study, indicating that the mutant was capable of persistence. Interestingly, detection of spirochetes in the blood of SCID mice was also delayed, resulting in statistically significant differences between the number of positive mice infected with *Bh*::Comp versus the wild type in the first 2 days post inoculation. These results suggest that the mutant displays deficiencies in migration to the bloodstream and/or a reduced in vivo growth rate. Regardless, delayed detection of *Bh*::Comp in the bloodstream of SCID mice suggests that the similar phenotype during infection of C3H mice is likely not a result of the host's adaptive immune response.

**Table 3:** Infectivity and persistence of *Borrelia hermsii* complemented mutants in C3H and SCID mice

Day post inoculation	C3H mice infected with				SCID mice infected with			
	WT	*Bh*::Comp	*Bh*::UHS_AS_	*Bh*::DHS_ΔIR_	WT	*Bh::Comp*	*Bh*::UHS_AS_	*Bh*::DHS_ΔIR_
1	4/5^a^	0/5	5/5	4/5	5/5	0/5**	5/5	3/5
2	4/5	0/5	5/5	5/5	5/5	0/5**	5/5	5/5
3	5/5	0/5^b^**	5/5	4/5	5/5	3/5	5/5	5/5
4	5/5	4/5	1/5*	0/5**	5/5	5/5	5/5	5/5
5	5/5	3/5	0/5**	0/5**	5/5	5/5	5/5	5/5
6	5/5	5/5	0/5**	0/5**	5/5	5/5	5/5	5/5
7	5/5	5/5	0/5**	0/5**	5/5	5/5	5/5	5/5
8	5/5	5/5	0/5**	0/5**	5/5	5/5	5/5	5/5
9	5/5	5/5	0/5**	0/5**	5/5	5/5	5/5	5/5
10	5/5	5/5	0/5**	0/5**	5/5	5/5	5/5	5/5

SCID, severe combined immunodeficient; WT, wild type; DHS, downstream homology sequence.

a Value listed corresponds to the number of spirochete positive mice/number tested.

b Statistically significant difference between mutant and wild‐type *B. hermsii* control groups denoted by asterisks as determined by Fisher's exact test. (**p < .01, *p < .05).

**Figure 7:**
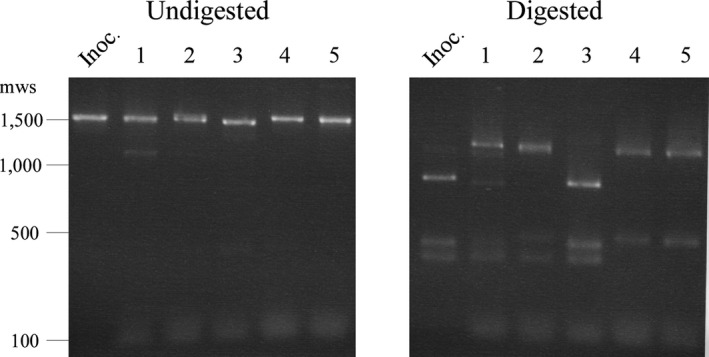
The *Bh*::Comp *vmp*_*Ex*_ locus undergoes genetic recombination by day 10 post inoculation in C3H mice. The *vmp*_*Ex*_ locus of *Bh*::Comp was PCR amplified from the starting inoculum (Inoc.), and from spirochetes recovered from C3H mice on day 10 post inoculation. The resultant PCR products are shown undigested, and digested with the restriction enzyme *Hind*III. Mouse number is indicated numerically at top; sizes of selected molecular weight standards (mws) of 1 kb DNA Ladder (New England Biolabs) are shown at left in base pairs

While it is clear from the murine infection results that *Bh*::Comp is able to persist throughout the 10‐day study, it was necessary to verify that antigenic switching had occurred in the complemented *vmp*_*Ex*_ locus. To achieve this, *vmp*_*Ex*_ was PCR amplified from the starting inoculum of *Bh*::Comp, and from spirochetes recovered from all five C3H mice at day 10 post inoculation. The resultant PCR products were digested with the restriction enzyme *Hind*III, and compared to the *vmp*_*Ex*_ locus in the starting inoculum by gel electrophoresis (Figure 7). The results demonstrated that the restriction fragment profile of the *vmp*_*Ex*_ locus from mice 1, 2, 4, and 5 were different on day 10 from the starting inoculum. To further confirm antigenic switching in *Bh*::Comp, the same *vmp*_*Ex*_ PCR products from spirochetes recovered on day 10 were submitted for sequencing followed by BLAST analysis. Spirochetes from mice 2, 4, and 5 had switched to VlpA18, while those from mouse three switched to VlpC19 (Data S1 depicts sequencing and alignment results for mice 3 and 4). Additionally, spirochetes from mouse 2 were also detected to switch to VlpD17. No results were obtained from mouse 1 due to a sequencing failure. Together, the results from the restriction fragment analysis and sequencing demonstrate that *Bh*::Comp is capable of antigenic variation and persistence during infection of an immunocompetent host.

### 3.5 Disruption of the UHS results in immune clearance

In order to determine the importance of the UHS in antigenic switching, the *Bh*::UHS_AS_ clone was used to infect mice and monitored for its ability to establish persistent infection. Unfortunately, this mutant was found to lack Vmp expression despite our best efforts to leave the transcriptional start site and ribosomal binding site in its native form (see Figure 5b). However, because VmpE antigenic variation in *B. hermsii* is known to occur as a result of genetic rearrangement, antigenic switching was predicted to occur even in the absence of initial Vmp expression. Therefore, the *Bh*::UHS_AS_ mutant was inoculated into groups of five C3H or SCID mice as described previously. Blood was collected from each mouse every 24 hr and monitored for the presence of spirochetes by direct microscopy or blood culture. The results revealed that *Bh*::UHS_AS_ is cleared in all immunocompetent mice by day 5 post inoculation, but persists in all immunodeficient SCID mice throughout the 10‐day experiment (Table 3). Persistence in SCID mice demonstrates the absence of inherent defects in infectivity, and reveals that the clearance of *Bh*::UHS_AS_ in C3H mice is a direct result of the host's immune response. Statistically significant differences in the number of *Bh*::UHS_AS_ positive mice compared to wild‐type *B. hermsii* was found from day 4 through 10 post inoculation. Interestingly, the results from *Bh*::UHS_AS_ and the *vmp*_*Ex*_ deletion mutant, *Bh*∆*vmp*_*Ex*_, closely resembled one another with no statistically significant differences in the number of positive mice between the two mutants at any time point. The murine *Bh*::UHS_AS_ infection results indicate that the mutant is incapable of persisting in immunocompetent mice. However, whether clearance of *Bh*::UHS_AS_ is due to defective *vmp*_*Ex*_ recombination or the absence of Vmp expression is unknown.

To assess whether the clearance of *Bh*::UHS_AS_ was due to the absence of antigenic switching, *vmp*_*Ex*_ was amplified from spirochetes recovered from C3H mice on the last day of positive infection in all mice (day 3) in order to subject the PCR amplicons to restriction fragment length polymorphism (RFLP) analysis. Not only did this approach fail to detect antigenic switching in *Bh*::UHS_AS_, switching was also not detected in the wild‐type spirochetes recovered from this early time point (Data S2). This finding was corroborated by an additional, independent experiment in which five C3H mice were inoculated with the wild‐type strain or *Bh*::UHS_AS_ and monitored for spirochetemia for 3 days. Once again, antigenic switching could not be detected by either RFLP analysis or DNA sequencing in *Bh*::UHS_AS_ nor wild‐type spirochetes recovered on day 3 post inoculation.

**Figure 8:**
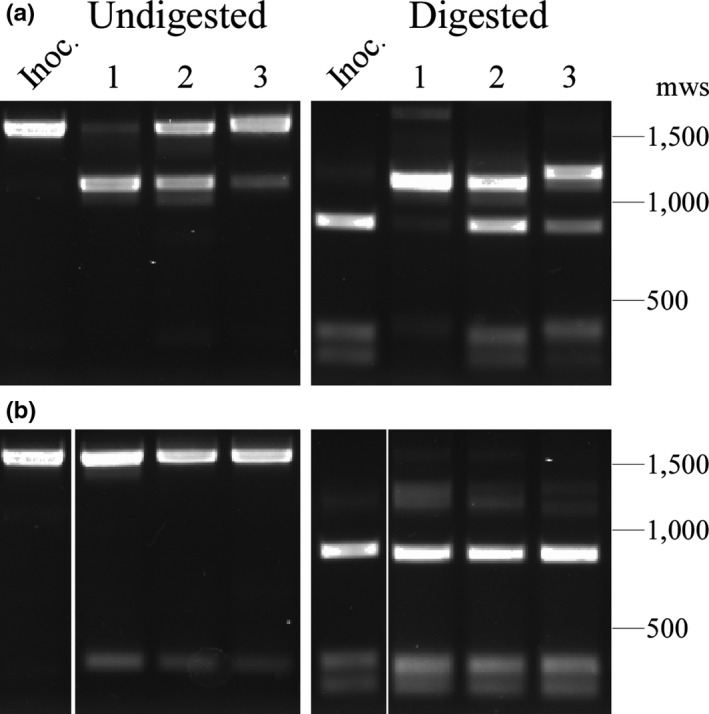
Wild‐type *Borrelia hermsii*, but not *Bh*::UHS_AS_, undergoes antigenic variation by day 10 post inoculation in severe combined immunodeficient (SCID) mice. The *vmp*_*Ex*_ locus of wild‐type *B. hermsii* (panel a) and *Bh*::UHS_AS_ (panel b) was PCR amplified from the starting inoculum (Inoc.), and from spirochetes recovered from SCID mice on day 10 post inoculation. The resultant PCR products are shown undigested, and digested with *Hind*III. Mouse number is indicated numerically at top; sizes of selected molecular weight standards (mws) of 1 kb DNA ladder (New England Biolabs) are shown at right in base pairs

While the mechanisms that regulate the genetic recombination of the *vmp*_*Ex*_ locus are unknown, it has been shown that antigenic switching occurs spontaneously in culture, and thus presumably, in SCID mice (Stoenner et al., 1982). Therefore, as a final attempt to verify the absence of antigenic switching, the experiment was independently repeated twice more with an additional two groups of three SCID mice inoculated with either wild‐type *B. hermsii* or *Bh*::UHS_AS_ (six mice total for each strain). On day 10 post inoculation, blood was collected from each SCID mouse for verification of spirochetemia, and to determine whether spirochetes had undergone genetic rearrangement by RFLP analysis and sequencing of *vmp*_*Ex*_. Both strains were persistent in the blood of all six SCID mice at day 10 post inoculation, but none of the *Bh*::UHS_AS_ spirochetes from any of the six mice had undergone *vmp*_*Ex*_ recombination (Data S3). Conversely, in wild‐type‐infected mice, the *vmp*_*Ex*_ site from spirochetes recovered from 5 of 6 mice revealed a mixed population of the infecting *vmp* variant and a switched variant (Data S4). No antigenic switching was detected in spirochetes from the remaining mouse infected with wild type. The detection of genetic recombination in spirochetes from 5/6 wild‐type‐infected SCID mice compared to 0/6 in *Bh*::UHS_AS_‐infected mice is statistically significant (*p* = .02). Finally, to further verify the absence of antigenic switching, the *vmp*_*Ex*_ amplicons of spirochetes recovered from the blood of three of the SCID mice infected with wild‐type *B. hermsii* or *Bh*::UHS_AS_ on day 10 post inoculation were digested with HindIII, and restriction fragment profiles were compared to the *vmp*_*Ex*_ locus of the infecting inoculum. The results similarly revealed that *Bh*::UHS_AS_ did not undergo antigenic variation over the course of 10 days in SCID mice, while wild‐type *B. hermsii* does (Figure 8). Overall, the data suggest that regardless of Vmp expression, *Bh*::UHS_AS_ is incapable of antigenic switching, lending support to the importance of the UHS for genetic recombination at *vmp*_*Ex*_.

### 3.6 Deletion of the DHS‐resident inverted repeat results in immune clearance

As previously mentioned, a 31 bp IR sequence is found within the DHS between positions 47 and 77 (Dai et al., 2006). IR sequences can form secondary stem‐loop structures in DNA, and these structures are known to be recombinogenic. To evaluate the importance of the IR sequence found within the *vmp*_*Ex*_ DHS for antigenic switching, groups of five C3H and SCID mice were infected with *Bh*::DHS_ΔIR_ as described above. To monitor for the presence of spirochetes, blood was collected from each mouse every day for 10 days post inoculation, and examined by direct microscopy or blood culture. The results showed that *Bh*::DHS_ΔIR_ is able to initially infect immunocompetent C3H mice, but is cleared in all five mice by day 4 post inoculation (Table 3). Statistically significant differences were found between the numbers of C3H mice infected with *Bh*::DHS_ΔIR_ compared to wild‐type *B. hermsii* from day 4 through 10 post inoculation. When compared with *Bh*∆*vmp*_*Ex*_, no statistically significant differences in the number of positive mice at any time point was found. Importantly, *Bh*::DHS_ΔIR_ is able to establish persistent infection in SCID mice (Table 3), demonstrating that the clearance of the mutant in SCID mice is a direct result of the host's immune response, and not some other defect in infectivity.

Finally, because wild‐type *B. hermsii* was shown to undergo antigenic switching in the absence of immune pressure, *vmp*_*Ex*_ amplicons of *Bh*::DHS_ΔIR_ recovered from five SCID mice on day 10 post inoculation were also sequenced and subjected to RFLP. Both RFLP and sequencing results revealed that all *vmp*_*Ex*_ sites from the *Bh*::DHS_ΔIR_ spirochetes recovered from the SCID group matched the *vmp* variant present in the infecting inoculum, further suggesting that this mutant is unable to antigenically vary (Data S5). Compared to wild‐type *B. hermsii*, where spirochetes recovered from 5/6 SCID mice had undergone antigenic switching by day 10 post inoculation (described above), the finding that switching was not detected in any of the five SCID mice infected with *Bh*::DHS_ΔIR_ is statistically significant (*p* = .02). Combined, the data from both restriction fragment analysis and sequencing suggest that the DHS‐resident IR is essential for antigenic switching, and thus is critical for persistence in the immunocompetent host.

## 4 DISCUSSION

While previous studies have implicated the importance of the UHS and DHS for *vmp*_*Ex*_ recombination (Barbour, Burman, et al., 1991; Dai et al., 2006; Kitten & Barbour, 1990), no studies have investigated the requirement of these elements for persistence using direct, mutational analysis. The results herein describe a targeted deletion and *in cis* complementation approach to establish the role of the UHS and DHS in antigenic switching. To our knowledge, these techniques have not been applied to any of the relapsing fever *Borrelia spp*. thus far. Using these methods, the findings of Raffel et al., 2014, have been corroborated by demonstrating that the absence of Vmp expression results in the inability of *B. hermsii* to persist in the immunocompetent mammalian host. The results in the present study expand on those findings by demonstrating that deletion of the entire *vmp*_*Ex*_ locus, and thus obliteration of any possibility for antigenic switching, results in immune clearance of spirochetes. We further report the successful complementation of a switchable *vmp*_*Ex*_ onto lpE27, and demonstrate that this mutant is capable of persistence during murine infection. When the UHS is disrupted, or the DHS‐resident IR deleted, the mutant *B. hermsii* are no longer capable of persistence. These results are the first to verify the importance of these elements for Vmp expression or antigenic switching, respectively, and serve to further characterize the underlying mechanism for the gene conversion event that is essential for the pathogenesis and life cycle of *B. hermsii*.

**Figure 9:**
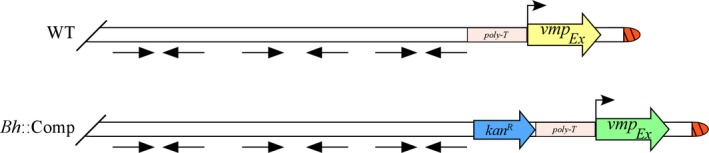
Schematic of the lpE27 plasmids in wild‐type *Borrelia hermsii* and *Bh*::Comp. The poly‐T run, inverted repeat sequences (depicted by arrows), *vmp*_*Ex*_ promoter (*p*, arrow), and *vmp*_*Ex*_ are depicted in wild‐type (WT) and complemented strains (*Bh*::Comp). A kanamycin resistance (*kan* ^R^) selection marker is shown in *Bh*::Comp. Schematic is not to scale

Upstream of the *vmp*_*Ex*_ promoter lies a 13 bp run of T‐residues in the *B. hermsii* strain DAH (Barbour, Burman, et al., 1991; Barbour, Carter, et al., 1991). Within the next approximately 6 kb immediately upstream of the poly‐T tract lie three IR sequences, each ~1 kb, that form putative secondary stem‐loop structures (Barbour, Carter, et al., 1991). Together, the poly‐T run and IR sequences are proposed to serve some role in the regulation of Vmp expression, or perhaps in *vmp*_*Ex*_ recombination, because both are only found in the *B. hermsii* genome upstream of the *vmp*_*Ex*_ promoter on lpE27 (Barbour, Carter, et al., 1991; Sohaskey, Zuckert, & Barbour, 1999). In fact, it has been demonstrated that the *B. hermsii* poly‐T run enhances transcription of a reporter construct in *B. burgdorferi*; no role for the IR sequences have been elucidated to date (Sohaskey et al., 1999). While the results herein did not seek to establish a role for the 5′ IR sequences, it is interesting that the complemented mutant, *Bh*::Comp, was able to undergo *vmp*_*Ex*_ recombination, despite a physical separation of 1,169 bp between the poly‐T run and the most proximal IR due to insertion of the *kan* ^R^ cassette (Figure 9). In wild‐type *B. hermsii*, the most 3′ IR begins only 1 bp from the poly‐T sequence (Barbour, Burman, et al., 1991; Barbour, Carter, et al., 1991). These findings suggest that the poly‐T and IR sequences do not exert their influence on *vmp*_*Ex*_ by spatial proximity, if they regulate Vmp expression and/or recombination at all.

For the *Bh*::UHS_AS_ mutant, the major conclusion that can be drawn from the results is that some element in this region, other than the transcriptional start site and ribosomal binding site, is required for transcription or translation of *vmp*_*Ex*_. The −10 and −35 promoter elements lie outside of the UHS boundaries and were not altered, thus whatever sequence is required for Vmp expression seems to lie within the UHS. One possibility may be the conserved 4‐mer and 6‐mer palindromes depicted in Figure 4. Conceivably, if antigenic variation were possible in *Bh*::UHS_AS_, it cannot be excluded that a gene conversion event would replace the defective UHS with a functional UHS that would restore Vmp surface expression *in vivo*. The failure to detect any *vmp*_*Ex*_ switching in *Bh*::UHS_AS_ after 10 days of infection in SCID mice lends support to the possibility that disruption of the UHS results in an absolute obliteration of *vmp*_*Ex*_ gene conversion, particularly in light of the finding that wild‐type *B. hermsii* switches in SCID mice by day 10 post inoculation. A caveat, however, is that it is not currently known whether the gene conversion process requires *vmp*_*Ex*_ transcription and/or translation. Moreover, further caution must be taken when interpreting the *Bh*::UHS_AS_ *vmp*_*Ex*_ sequence analyses from SCID mice as it is unknown how the alterations in the UHS and DHS elements affect the kinetics of switching. Here, switching may have occurred, but at a lower rate than wild type and therefore, was not detected. Future investigations can take advantage of the targeted deletion/*in cis* complementation technique to evaluate which sequences within the UHS are specifically required for Vmp expression.

The data presented herein indicate that the IR sequence is required for the recombination event that leads to relapses. This requirement provides a partial explanation for the conservation of the archived DHS sites that are scattered throughout the *B. hermsii* genome. A major question remains, however, and that is, “is the DHS‐resident IR structure sufficient for *vmp*_*Ex*_ recombination?” Based on these results, it is unclear if the *Bh*::DHS_ΔIR_ mutant did not persist in immunocompetent mice because it lacked the secondary hairpin structure characteristic of IR sequences, or if deleting the IR destroyed enough homology within the DHS that genetic recombination was no longer possible. To address this, it will be interesting to generate a mutant with the DNA homology in the DHS destroyed while leaving the IR intact, and another with only half of the IR sequence disrupted to obliterate secondary structure formation.

What role do IR sequences, such as the 31 bp DHS‐resident IR, serve in recombination? The secondary stem‐loop structures that IR's form in DNA are known to induce genetic instability (reviewed in Wang & Vasquez, 2006; Zhao, Bacolla, Wang, & Vasquez, 2010). Indeed, secondary DNA structures are associated with recombination in a number of prokaryotic and eukaryotic organisms. The exact mechanisms vary, but these secondary structures may stall replication forks, leading to collapse of the fork and double‐stranded breaks. Another possibility is that stem‐loop structures attract DNA repair proteins that recognize the secondary structure as “damaged,” and induce double‐stranded breaks (Zhao et al., 2010). While the exact mechanism underlying the non‐reciprocal gene conversion event that results in antigenic variation in *B. hermsii* remains unknown, the results presented herein verify the importance of the DHS‐resident IR for efficient *vmp*_*Ex*_ recombination.

In addition to confirming the requirement of the DHS‐resident IR for antigenic switching, the data provide evidence for the importance of the UHS in Vmp expression and suggest that the homology in the UHS may be an essential feature for antigenic variation. Using the targeted deletion/*in cis* complementation technique, ongoing studies will investigate other features of the UHS and DHS elements, such as the role for the conserved 4‐mer and 6‐mer palindromes as identified by Dai et al. (2006). Nevertheless, the results from this study have revealed further insight for the mechanism of antigenic variation at the molecular level, and have provided new tools for the genetic study of *B. hermsii*.

## CONFLICT OF INTEREST

None declared.
