# Supplementary material for: Cis‐acting DNA elements flanking the variable major protein expression site of Borrelia hermsii are required for murine persistence
Source: Microbiologyopen. 2017 Dec 17;7(3):e00569. doi: 10.1002/mbo3.569 (PMC6011951; doi:10.1002/mbo3.569)
Supplement: Supplementary file 1 [file MBO3-7-e00569-s001.pdf]

Primer	Sequence (5' to 3')[a]
P237	A**TATAATTTGATATTAGTACAAATCCC**CCGATATCGCCGTGGCG[b]
P259	A**TATAATTTGATATTAGTACAAATCCC**CCACTAGTAACGGCCGCC[b]
P274	GTGCCCGGATCCTTAAACATCATATCCAAAAATTAAAGCTAAATA
P275	CCGCGGCGCATATGTCGTTGAAAGTTAATATAAAAA
P395	ATTGGCGCGCCAAGGATAGCAGAAGTACTCTTTCCTACGCC
P396	GAACTCGAGCCTAACGCAACGAGGGCTGA
P422	TAGGTTTAAACCCAGCACACTGGCGGC
P423	TAGAGTACT CTCGAGGCCACGTTGTGTCTCAAAATCTCTG
P424	TAGAGTACT GGCGCGCCCGTGAGTTTTCGTTCCACTGAGC
P425	TAGGTTTAAACTGCCCGCTTTCCAGTCG
P626	ATTGGCGCGCCGCTCCTTTAGCAACAGTGACATAATCAGC
P627	GAACTCGAGCTTTAGCCATTCTCTTAATGCTATAGCTCTTGC
P541	TAGGCTAGCGCAAGAGCTATAGCATTAAGAGAATGGCTAAAG
P542	TAGTGCGCATCCCCAGTGCTTCTTTTCCTTCC
P233	ATTCATATGAACACCCTCTATATCACAAATT
P537	GTCGTTAGTATTTTTTTGGAGAGAAGTATC
P854	GTTCTGTTCAGGACTTATAGGAGTATC
P855	CCATGTTGGAATTTAATCGC
P558	CATATGAATAGCGGTGGGGTTGC
P559	GGATCCCTAGTTACTAGTAGTAGCTTTAGGGATATTCTGC

[a] Restriction enzyme sites underlined.

[b] Half of the *B. hermsii* lpE27 replicated telomere in bold.
