# Supplementary material for: Cis‐acting DNA elements flanking the variable major protein expression site of Borrelia hermsii are required for murine persistence
Source: Microbiologyopen. 2017 Dec 17;7(3):e00569. doi: 10.1002/mbo3.569 (PMC6011951; doi:10.1002/mbo3.569)
Supplement: Supplementary file 2 [file MBO3-7-e00569-s002.pdf]

Strain	β_1[a]	95% CI	p-value[b]
Wild type	0.032	0.026-0.039	-
*Bh*Δ*vmp*$_{EX}$	0.029	0.021-0.037	0.37
Bh::Comp	0.032	0.030-0.034	0.99
Bh::UHS$_{AS}$	0.033	0.031-0.036	0.77
Bh::DHS$_{\Delta IR}$	0.029	0.022-0.035	0.30

[a] Slope was calculated for the period of 72 hours of exponential growth (days 1-4 post subculture)

[b] p-value of slopes compared to the wild type strain
