# Supplementary material for: Cis‐acting DNA elements flanking the variable major protein expression site of Borrelia hermsii are required for murine persistence
Source: Microbiologyopen. 2017 Dec 17;7(3):e00569. doi: 10.1002/mbo3.569 (PMC6011951; doi:10.1002/mbo3.569)
Supplement: Supplementary file 3 [file MBO3-7-e00569-s003.pdf]

Strain	Mean Maximum Cell Density[a]	95% Confidence Interval	p-value[b]
WT	3.4×10^8	$1.8 \times 10^8 – 5.1 \times 10^8$	–
Bh::Comp	2.6×10^8	$2.1 \times 10^8 – 3.0 \times 10^8$	0.37
Bh::UHS_{AS}	2.4×10^8	$1.4 \times 10^8 – 3.5 \times 10^8$	0.37
Bh::$DHS_{\Delta IR}$	3.5×10^8	$2.7 \times 10^8 – 4.4 \times 10^8$	0.96

[a] Cell density is equivalent to spirochetes per mL of blood

[b] p-value of mean maximum cell density compared to the wild type strain
