# Supplementary material for: Cis‐acting DNA elements flanking the variable major protein expression site of Borrelia hermsii are required for murine persistence
Source: Microbiologyopen. 2017 Dec 17;7(3):e00569. doi: 10.1002/mbo3.569 (PMC6011951; doi:10.1002/mbo3.569)
Supplement: Supplementary file 4 [file MBO3-7-e00569-s004.pdf]

Mouse 3

```
                          1                                                                                                  100
Bh::Comp vmpEx Inoc     (1) ---------GCTAGCGCAAGAGCTATAGCATTAAGAGAATGGCTAAAGGTGATAAATTTGATTTTTTTTTTTTTAACTTTGTAAACTTTGAAAGTTGAGG
Recovered vmpEx C3H     (1) TAGGCCGGCACTAGTGCAAGAGCTATAGCATTAAGAGAATGGCTAAAGGTGATAAATTTGATTTTTTTTTTTTTAACTTTGTAAACTTTGAAAGTTGAGG
                          101                                                                                                200
Bh::Comp vmpEx Inoc    (92) TATAATGCTAATGCATAAGTTAAAAGGAGGCACGTAAAAAAATGAGAAAAAGAATAAGTGCAATAATAATGACTTTATTTATGGTATTAGTAAGCTGTAA
Recovered vmpEx C3H   (101) TATAATGCTAATGCATAAGTTAAAAGGAGGCACGTAAAAAAATGAGAAAAAGAATAAGTGCAATAATAATGACTTTATTTATGGTATTAGTAAGCTGTAA
                          201                                                                                                300
Bh::Comp vmpEx Inoc   (192) TAGCGGTGGGGTTGCGGAAGACCCTCAGAGTAAATTTTTAAAGTCAGCAATAGACTTAGGTAATGATTTTTTAAATGTGTTTACATCATTTGGAGATATA
Recovered vmpEx C3H   (201) TAGCGGTGGGGTTGCGGAAGACCCTCAGAGTAAATTTTTAAAGTCAGCAATAGACTTAGGTAATGATTTTTTAAATGTGTTTACATCATTTGGAGATATA
                          301                                                                                                400
Bh::Comp vmpEx Inoc   (292) GTTTCCAAGGTATTAGGTTTTAGTACAGAGACAAAAAAGTCTGATGTTGGGGCTTATTTTAAGACAATACAAGATACTATACAAGGCACTAAGGACAAGC
Recovered vmpEx C3H   (301) GTTTCCAAGGTATTAGGTTTTAGTACAGAGACAAAAAAGTCTGATGTTGGGGCTTATTTTAAGACAATACAAGATACTATACAAGGCACTAAGGACAAGC
                          401                                                                                                500
Bh::Comp vmpEx Inoc   (392) TTAATAAAATTGTTACTGACATGAAGAGAGAAGGAAATCCTAATGCTTCTGCAACTGAGACTGCGGTAAAAACACTAATTGATAATACTCTTGATAAGAT
Recovered vmpEx C3H   (401) TTAATAAAATTGTTACTGACATGAAGAGAGAAGGAAATCCTAATGCTTCTGCAACTGAGACTGCGGTAAAAACACTAATTGATAATACTCTTGATAAGAT
                          501                                                                                                600
Bh::Comp vmpEx Inoc   (492) AATAGAAGGTGCTGAGACTGCAAGTGAGGCTATTGGTGATGCTGGTGACCCAATTGGTAATGTTGCTGCTGGTGGTGCTGGTGCGGGTACAGGTGCTATT
Recovered vmpEx C3H   (501) AATAGAAGGTGCTAAGACCGTTAGTGATGCTATTGGAGATGCTAGTGACCCAATTGCTAATGTTGCTGCTCAGAATGCTGCTG---------GTGCTGCT
                          601                                                                                                700
Bh::Comp vmpEx Inoc   (592) GGGGATGGTGTTGATAATCTAATAAATGGAATTAAGGCAATTGTAGAAGTAGTACTTAAAGA---AGGGAATGCTGAGGCTGGAGATGGTAAAAAGGCCG
Recovered vmpEx C3H   (592) GGGACTGAAGTTGACAAACTAGTAAAAGGAATAAAGACAATTGTAGACGTGGTACTTAAAGGTGTAGGAAGTGCTAATGCTGGTGATGATAAAAAGGCTG
                          701                                                                                                800
Bh::Comp vmpEx Inoc   (689) ATGCTCT---TGGAGCAAGAGGTGCTAATGCTGGTGATGCAGGAAAG--TTATTTGGTAATACTGGTAATAATGGTGCTATTGATTCTGCAGATAATGCA
Recovered vmpEx C3H   (692) AAGATGGCAATACTGCAAGAACTGCTGCTGCTGGCGATGGTGAAGCGGGTAAATTATT--TACTGCTGGTGCAGGTGCTGCTGGTGATGCTAATAATGCA
                          801                                                                                                900
Bh::Comp vmpEx Inoc   (784) AAGAAAGCAGGTGCTGATGCAGCAAAAGCAGTAGGGGCAGTAACAGGTGCTGATATATTACAAGCTATTTCTAAAGATGGTGGTGATGCTGCTAAATTAG
Recovered vmpEx C3H   (790) AAAAAAGTTGCAGCTGATGCAGCAAAAGCAGTAGGAGCTGTAACTGGTGCTGACATATTACAAGCTATGATTAAAGATAATGGTGATGCTGCTAAATTAG
                          901                                                                                               1000
Bh::Comp vmpEx Inoc   (884) CTAAGAATAGTGCTACCGTTCAGGTGACTGGTGTTGCTGTTGATGTTAAAGATGCGGTTATAGCAGGAGGAATTGCACTCAGAGCAATGGCAAAGGGTGG
Recovered vmpEx C3H   (890) ------------CTACTGCTCAAAATGCTGGTGCTGCCCCT------AAAGATGGAGCTATTGCAGGAGGTATAGCTTTAAGAGTTATGGCAAAGGGTGG
                          1001                                                                                              1100
Bh::Comp vmpEx Inoc   (984) TAAATTTGCTAATGATAAGGATGCTGTTAATGCTGATGTTGTTACTGCAGTTAAAGGAGCAACAGTAAGTGCAGTAACTAAAGCACTAGATACATTAACT
Recovered vmpEx C3H   (972) TAAATTTGCTGGCCCTAG---TGCTGCTGCTGATGATGCTGTTACTGCAATTAAAGGAGCAGCAATAAGTTCAGTAACTAAAGCATTAGATACATTAACT
                          1101                                                                                              1200
Bh::Comp vmpEx Inoc  (1084) ATTGCAATAAGAAAAACAATTGACGCAGGCCTTAAAACAGTTAAAGAAGCTATGAAAATTAATGCTAATGATACTCCTATAACTCCTGAGCAGAATATCC
Recovered vmpEx C3H  (1069) ATTGCAATAAGAAAAACAATTGATGTGGGACTTAAAACGGTTAAAGAAGCTATGAAAATTAATGCTAATGATACTCCTATAACTCCTGAACAGAGCGCCC
                          1201                                                                                              1300
Bh::Comp vmpEx Inoc  (1184) CTAAAGCTACTACTAGTAACTAGTTAAGGATAAATATAAAGGATAAAGTCATTGTAAGGGAAAAGCTTTTCTTGTTTTTAATGCAGGAGTGTAGTTTCTC
Recovered vmpEx C3H  (1169) CTAAAGGCACTACTAATAACTAGTTAAGGATAAGTATAAAGGATAAAGTTATTGTAAGGGAAAAGCTTTTCTTGTTTTTAATGCAGGAGTGTAGTTTCTC
                          1301                                                                                              1400
Bh::Comp vmpEx Inoc  (1284) TGATTAAGTAAGCTGTAAGAGCAGGGAAAAATAAAGTCAAAAAGGAATAGGAAGCTAGGAGCGTAATGCTCTTAGCTTCTAATGTTATTTAGGGAGTGTT
Recovered vmpEx C3H  (1269) TGATTAAGTAAGCTGTAAGAGCAGGGAAAAATAAAGTCAAAAAGGAATAGGAAGCTAGGAGCGTAATGCTCTTAGCTTCTAATGTTATTTAGGGAGTGTT
                          1401                                                                                              1500
Bh::Comp vmpEx Inoc  (1384) TCTTTGTATATAAAATTGTTTATATGAGTAAAGATTGAATATAAATAATTGCAAGTATGATATTAAGAGTATGTTTTTATTGTAATCAAATAATTAATAC
Recovered vmpEx C3H  (1369) TCTTTGTATATAAAATTGTTTATATGAGTAAAGATTGAATATAAATAATTGCAAGTATGATATTAAGAGTATGTTTTTATTGTAATCAAATAATTAATAC
                          1501                                                                              1587
Bh::Comp vmpEx Inoc  (1484) TTTAAAAGTAAGCTAAATGTGTGGTAAGGGCAGCAAAAGGGAAATTGGGATAGATGTTGGAAGGAAAAGAAGCACTGGGGATGCGCA
Recovered vmpEx C3H  (1469) TTTAAAAGTAAGCTAAATGTGTGGTAAGGGCAGCAAAAGGGAAATTGGGATAGATGTTGGAAGGAAAAGAAGCACTGGGAGCCGGCC
```

Mouse 4

```
                              1                                                                                                  100
Bh::Comp vmpEx Inoc      (1) ---------GCTAGCGCAAGAGCTATAGCATTAAGAGAATGGCTAAAGGTGATAAATTTGATTTTTTTTTTTTTTAACTTTGTAAACTTTGAAAGTTGAGG
Recovered vmpEx C3H      (1) TAGGCCGGCACTAGTGCAAGAGCTATAGCATTAAGAGAATGGCTAAAGGTGATAAATTTGATTTTTTTTTTTTTTAACTTTGTAAACTTTGAAAGTTGAGG
                              101                                                                                                200
Bh::Comp vmpEx Inoc     (92) TATAATGCTAATGCATAAGTTAAAAGGAGGCACGTAAAAAAATGAGAAAAAGAATAAGTGCAATAAT-AATGACTT-----------TATTTATGGTATT
Recovered vmpEx C3H    (101) TATAATGCTAATGCATAAGTTAAAAGGAGGCACGTAAAAAAATGAGAAAAAGAATAAGTGCAATAATTAATAAGTTAAATATAAGTATAATGATGATGAT
                              201                                                                                                300
Bh::Comp vmpEx Inoc    (180) AGT--------------------------AAGCTGTAATAGCGG-------TGGGGTTGCGGAAGACCCTCAGAGTAAATTTTTAAAGTCAGCAATA---
Recovered vmpEx C3H    (201) AGTTGTTCTAATGATAGGATGTGGACAACAGGCAGTAGAAGCAGGGAAGGATGGCGCAGCAGCAGCTACAGGGGGAAGAAGTTTAAGTGAAGTACTAATG
                              301                                                                                                400
Bh::Comp vmpEx Inoc    (244) GACTTAGGTAATGATTTTTTAAATGTGTTTACATCATTTGGAGATATAGTTTCCAAGGTATTAGGTTTTAG------TACAGAGACAAAAAAGTCTGATG
Recovered vmpEx C3H    (301) GAAGTGGGAAAAAGTGCTGAGAATGCTTTTTATTCATTTATGGCTTTAGTCTCAGATACATTAGGCTTAAGAGTGACTAAAGATACAAAGAAGAATGAAG
                              401                                                                                                500
Bh::Comp vmpEx Inoc    (338) TTGGGGCTTATTTTAAGACAATACAAGATA--CTATACAAGGCACTA-----AGGACAAGCT-TAATAAAATTGTTACTGACATGAAGAGAGAAGGAAAT
Recovered vmpEx C3H    (401) TGGGAGGTTATTTTAACAGCCTAGGTGGTAAGCTTGGAAAAGCATCAGATGAATTAGAAGAAGTAGCAAAAAAGTCAGAAGTAGAAGGAGCTAAAGATGG
                              501                                                                                                600
Bh::Comp vmpEx Inoc    (430) CCTAAT-GCTTCTGCAACTGAGACTGCGGTAAAAACACTAATTGATAATACTCTTGATAAGATAATAGAAGGTGCTGAGACTGCAAGTGAGGCTATTGGT
Recovered vmpEx C3H    (501) ACCAATAGCTGTAGCAATTAGAGCCGCAGTTGATACA---GCTAAGACTACTTTAAGTACATTAAAAG--GGCATTTAGAATCCTTAAAAGGG-ATAGGT
                              601                                                                                                700
Bh::Comp vmpEx Inoc    (529) GATGCTGGTGACCCAATTGGTAATGTTGCTGCTGGTGGTGCTGGTGCGGGTACAGGTGCTATTGGGGATGGTGTTGATAATCTAATAAATGGAATTAAGG
Recovered vmpEx C3H    (595) GATG-------ACAAAGTAGTAG-GTTGG-GCAGAAAATGATCAACAAGGAATCAAACCAGCTGATGATGGATTGAATAAATTCCTTAATGCATTGCAGT
                              701                                                                                                800
Bh::Comp vmpEx Inoc    (629) CAATTGTAGAAGTAGTACTTAAAGAAGGGAATGCTGAGGCTGGAGATGGTAAAAAGGCCGATGCTCTTGGAGCAAGAGGTGCTAATGCTGGTGATGCAGG
Recovered vmpEx C3H    (686) CAATAGTAAAGGCAGCAACAGATGCAGGTGTTTTAGCACCAAAAGCAGGGAATACAACATTGACAGTAAATGGGGTAGATAATAAGGATGGTGCTAAGGT
                              801                                                                                                900
Bh::Comp vmpEx Inoc    (729) AA-AGTTATTTGGTAATACTGGTAATAATGGTGCTATTGATTCTGCAGATAATGCAAAGAAAGCAGGTGCTGATGCAGCAAAAGCAG---TAGGGGCAGT
Recovered vmpEx C3H    (786) ATTAGCTATA-GATAAACCAGGGGCAGCAGTAGGAGAAAAAGCATCATTAATAGTATCGGCAGTAAGTGGAGAGGAAATACTAGCATCAATAGTTGCGTC
                              901                                                                                                1000
Bh::Comp vmpEx Inoc    (825) AACAGGTGCTGATATATTACAAGCTATTTCTAAAGATGGTGGTGATGCTGCTA--AATTAGCTAAGAATAGTGCTACCGTTCAGGTGACTGGTGTTGCTG
Recovered vmpEx C3H    (885) AAAAGAAGGTGATCAAGCACTAGGAGCAGCTGCAGATGGAACTACAACTGCGATGAGTTTTGCAAAAGGAGGAACAAAGGACAACCTATCAA--ATGCAA
                              1001                                                                                               1100
Bh::Comp vmpEx Inoc    (923) TTGATGTTAAAGATGCGGTTATAGCAGGAGGAATTGCACTCAGAGCAATGGCAAAGGGTGGTAAATTTGCTAATGATAAGGATGCTGTTAATGCTGATGT
Recovered vmpEx C3H    (983) ATACACCAAAAGCAGCAGCAGTAGCAGGAGGAATAGCACTACGTTCCTTAGTTAAAGATGGTAAATTAGCTTCACATAATGAT------AATAGTGAA--
                              1101                                                                                               1200
Bh::Comp vmpEx Inoc   (1023) TGTTACTGCAGTTAAAGGAGCAACAGTAAGTGCAGTAACTAAAGCACTAGATACATTAACTATTGCAATAAGAAAAACAATTGACGCAGGCCTTAAAACA
Recovered vmpEx C3H   (1075) ----AAAGCAGTACAAGCAGCAGGAGTAATTGCAGCAAATAAGTTATTAGTATCAGTAGAAGATCTAATTAAGAAGACAGTAAAGAATGTTCTTGAGAAA
                              1201                                                                                               1300
Bh::Comp vmpEx Inoc   (1123) GTTAAAGAAGCTATGAAAATTAATGCTAATGATACTCCTATAACTCCTGAGCAG-AATATCCCTAAAGCTACTACTAGTAACTAGTTAAGGATAAATATA
Recovered vmpEx C3H   (1171) GCAAAAGAA---AAAATAGATAAAGCAAGAG---CTCCAAAAGCAACAGGTCAGCAATA---AGATAGATAGTTAGATTAATTACTTAAAAGTAAGAGTA
                              1301                                                                                               1400
Bh::Comp vmpEx Inoc   (1222) AAGGATAAAGTCATTGTAAGGGAAAAGCTT--TTCT---TGTTTTTAATGCAGGAGT-GTAGTTTCTCTGA---------TTAAGTAAGCTGTAAGAGCA
Recovered vmpEx C3H   (1262) GAAGGCAATCTTAGAGATGCGTCTCAGATGCCTTCTATATTGTTGTAATGAAAAATAAGAAGTTATTATAAGATAAGTTATTAAGTAAGCTGTAAGAGCA
                              1401                                                                                               1500
Bh::Comp vmpEx Inoc   (1307) GGGAAAAATAAAGTCAAAAAGGAATAGGAAGCTAGGAGCGTAATGCTCTTAGCTTCTAATGTTATTTAGGGAGTGTTTCTTTGTATATAAAATTGTTTAT
Recovered vmpEx C3H   (1362) GGGAAAAATAAAGTCAAAAAGAAATAGGAAGCTAGGAGCGTAATGCTCTTAGCTTCTAATGTTATTTAGGGAATGTTTATTTGTATATAAAATTGTTTAT
                              1501                                                                                               1600
Bh::Comp vmpEx Inoc   (1407) ATGAGTAAAGATTGAATATAAATAATTGCAAGTATGATATTAAGAGTATGTTTTTATTGTAATCAAATAATTAATACTTTAAAAGTAAGCTAAATGTGTG
Recovered vmpEx C3H   (1462) ATGAGTAAAGATTGAATATAAATAATTGCAAGTGTGATATTAAGAGTATGTTTTTATTGTAATCAAATAATTAATACTTTAAAAGTAAGCTAAATGTGTG
                              1601                                                                        1678
Bh::Comp vmpEx Inoc   (1507) GTAAGGGCAGCAAAAGGGAAATTGGGATAGATGTTGGAAGGAAAAGAAGCACTGGGGATGCGCA--------------
Recovered vmpEx C3H   (1562) GTAAGGGCAGCAAAAGGGAAATTGGGATAGATGTTGGAAGGAAAAGAAGCACTGGGAGCCGGCCTAATCTTCTAGAAG
```
