# Supplementary material for: Cis‐acting DNA elements flanking the variable major protein expression site of Borrelia hermsii are required for murine persistence
Source: Microbiologyopen. 2017 Dec 17;7(3):e00569. doi: 10.1002/mbo3.569 (PMC6011951; doi:10.1002/mbo3.569)
Supplement: Supplementary file 5 [file MBO3-7-e00569-s005.pdf]

1 100

Bh::UHS$_{AS}$ *vmp*$_{Ex}$ Inoc (1) TAGGCTAGCGCAAGAGCTATAGCATTAAGAGAATGGCTAAAGGTGATAAATTTGATTTTTTTTTTTTTAACTTTGTAAACTTTGAAAGTTGAGGTATAAT

Recovered *vmp*$_{Ex}$ C3H (1) --CTTTGTAAACTTTGAAAGTTGAGGTATAAT

101 200

Bh::UHS$_{AS}$ *vmp*$_{Ex}$ Inoc (101) TATGACGCAACATTAAGTAGGAGGAAAGCATTTCAATGAGGAAGAGGATCAGCGCGATCATAATGACTTTATTTATGGTATTAGTAAGCTGTAATAGCGG

Recovered *vmp*$_{Ex}$ C3H (31) TATGACGCAACATTAAGTAGGAGGAAAGCATTTCAATGAGGAAGAGGATCAGCGCGATCATAATGACTTTATTTATGGTATTAGTAAGCTGTAATAGCGG

201 300

Bh::UHS$_{AS}$ *vmp*$_{Ex}$ Inoc (201) TGGGGTTGCGGAAGACCCTCAGAGTAAATTTTTAAAGTCAGCAATAGACTTAGGTAATGATTTTTTAAATGTGTTTACATCATTTGGAGATATAGTTTCC

Recovered *vmp*$_{Ex}$ C3H (131) TGGGGTTGCGGAAGACCCTCAGAGTAAATTTTTAAAGTCAGCAATAGACTTAGGTAATGATTTTTTAAATGTGTTTACATCATTTGGAGATATAGTTTCC

301 400

Bh::UHS$_{AS}$ *vmp*$_{Ex}$ Inoc (301) AAGGTATTAGGTTTTAGTACAGAGACAAAAAAGTCTGATGTTGGGGCTTATTTTAAGACAATACAAGATACTATACAAGGCACTAAGGACAAGCTTAATA

Recovered *vmp*$_{Ex}$ C3H (231) AAGGTATTAGGTTTTAGTACAGAGACAAAAAAGTCTGATGTTGGGGCTTATTTTAAGACAATACAAGATACTATACAAGGCACTAAGGACAAGCTTAATA

401 500

Bh::UHS$_{AS}$ *vmp*$_{Ex}$ Inoc (401) AAATTGTTACTGACATGAAGAGAGAAGGAAATCCTAATGCTTCTGCAACTGAGACTGCGGTAAAAACACTAATTGATAATACTCTTGATAAGATAATAGA

Recovered *vmp*$_{Ex}$ C3H (331) AAATTGTTACTGACATGAAGAGAGAAGGAAATCCTAATGCTTCTGCAACTGAGACTGCGGTAAAAACACTAATTGATAATACTCTTGATAAGATAATAGA

501 600

Bh::UHS$_{AS}$ *vmp*$_{Ex}$ Inoc (501) AGGTGCTGAGACTGCAAGTGAGGCTATTGGTGATGCTGGTGACCCAATTGGTAATGTTGCTGCTGGTGGTGCTGGTGCGGGTACAGGTGCTATTGGGGAT

Recovered *vmp*$_{Ex}$ C3H (431) AGGTGCTGAGACTGCAAGTGAGGCTATTGGTGATGCTGGTGACCCAATTGGTAATGTTGCTGCTGGTGGTGCTGGTGCGGGTACAGGTGCTATTGGGGAT

601 700

Bh::UHS$_{AS}$ *vmp*$_{Ex}$ Inoc (601) GGTGTTGATAATCTAATAAATGGAATTAAGGCAATTGTAGAAGTAGTACTTAAAGAAGGGAATGCTGAGGCTGGAGATGGTAAAAAGGCCGATGCTCTTG

Recovered *vmp*$_{Ex}$ C3H (531) GGTGTTGATAATCTAATAAATGGAATTAAGGCAATTGTAGAAGTAGTACTTAAAGAAGGGAATGCTGAGGCTGGAGATGGTAAAAAGGCCGATGCTCTTG

701 800

Bh::UHS$_{AS}$ *vmp*$_{Ex}$ Inoc (701) GAGCAAGAGGTGCTAATGCTGGTGATGCAGGAAAGTTATTTGGTAATACTGGTAATAATGGTGCTATTGATTCTGCAGATAATGCAAAGAAAGCAGGTGC

Recovered *vmp*$_{Ex}$ C3H (631) GAGCAAGAGGTGCTAATGCTGGTGATGCAGGAAAGTTATTTGGTAATACTGGTAATAATGGTGCTATTGATTCTGCAGATAATGCAAAGAAAGCAGGTGC

801 900

Bh::UHS$_{AS}$ *vmp*$_{Ex}$ Inoc (801) TGATGCAGCAAAAGCAGTAGGGGCAGTAACAGGTGCTGATATATTACAAGCTATTTCTAAAGATGGTGGTGATGCTGCTAAATTAGCTAAGAATAGTGCT

Recovered *vmp*$_{Ex}$ C3H (731) TGATGCAGCAAAAGCAGTAGGGGCAGTAACAGGTGCTGATATATTACAAGCTATTTCTAAAGATGGTGGTGATGCTGCTAAATTAGCTAAGAATAGTGCT

901 1000

Bh::UHS$_{AS}$ *vmp*$_{Ex}$ Inoc (901) ACCGTTCAGGTGACTGGTGTTGCTGTTGATGTTAAAGATGCGGTTATAGCAGGAGGAATTGCACTCAGAGCAATGGCAAAGGGTGGTAAATTTGCTAATG

Recovered *vmp*$_{Ex}$ C3H (831) ACCGTTCAGGTGACTGGTGTTGCTGTTGATGTTAAAGATGCGGTTATAGCAGGAGGAATTGCACTCAGAGCAATGGCAAAGGGTGGTAAATTTGCTAATG

1001 1100

Bh::UHS$_{AS}$ *vmp*$_{Ex}$ Inoc (1001) ATAAGGATGCTGTTAATGCTGATGTTGTTACTGCAGTTAAAGGAGCAACAGTAAGTGCAGTAACTAAAGCACTAGATACATTAACTATTGCAATAAGAAA

Recovered *vmp*$_{Ex}$ C3H (931) ATAAGGATGCTGTTAATGCTGATGTTGTTACTGCAGTTAAAGGAGCAACAGTAAGTGCAGTAACTAAAGCACTAGATACATTAACTATTGCAATAAGAAA

1101 1200

Bh::UHS$_{AS}$ *vmp*$_{Ex}$ Inoc (1101) AACAATTGACGCAGGCCTTAAAACAGTTAAAGAAGCTATGAAAATTAATGCTAATGATACTCCTATAACTCCTGAGCAGAATATCCCTAAAGCTACTACT

Recovered *vmp*$_{Ex}$ C3H (1031) AACAATTGACGCAGGCCTTAAAACAGTTAAAGAAGCTATGAAAATTAATGCTAATGATACTCCTATAACTCCTGAGCAGAATATCCCTAAAGCTACTACT

1201 1300

Bh::UHS$_{AS}$ *vmp*$_{Ex}$ Inoc (1201) AGTAACTAGTTAAGGATAAATATAAAGGATAAAGTCATTGTAAGGGAAAAGCTTTTCTTGTTTTTAATGCAGGAGTGTAGTTTCTCTGATTAAGTAAGCT

Recovered *vmp*$_{Ex}$ C3H (1131) AGTAACTAGTTAAGGATAAATATAAAGGATAAAGTCATTGTAAGGGAAAAGCTTTTCTTGTTTTTAATGCAGGAGTGTAGTTTCTCTGATTAAGTAAGCT

1301 1400

Bh::UHS$_{AS}$ *vmp*$_{Ex}$ Inoc (1301) GTAAGAGCAGGGAAAAATAAAGTCAAAAAGGAATAGGAAGCTAGGAGCGTAATGCTCTTAGCTTCTAATGTTATTTAGGGAGTGTTTCTTTGTATATAAA

Recovered *vmp*$_{Ex}$ C3H (1231) GTAAGAGCAGGGAAAAATAAAGTCAAAAAGGAATAGGAAGCTAGGAGCGTAATGCTCTTAGCTTCTAATGTTATTTAGGGAGTGTTTCTTTGTATATAAA

1401 1500

Bh::UHS$_{AS}$ *vmp*$_{Ex}$ Inoc (1401) ATTGTTTATATGAGTAAAGATTGAATATAAATAATTGCAAGTATGATATTAAGAGTATGTTTTTATTGTAATCAAATAATTAATACTTTAAAAGTAAGCT

Recovered *vmp*$_{Ex}$ C3H (1331) ATTGTTTATATGAGTAAAGATTGAATATAAATAATTGCAAGTATGATATTAAGAGTATGTTTTTATTGTAATCAAATAATTAATACTTTAAAAGTAAGCT

1501 1576

Bh::UHS$_{AS}$ *vmp*$_{Ex}$ Inoc (1501) AAATGTGTGGTAAGGGCAGCAAAAGGGAAATTGGGATAGATGTTGGAAGGAAAGAAGCACTGGGGATGCGCATAG

Recovered *vmp*$_{Ex}$ C3H (1431) AAATGTGT--
