# Supplementary material for: Cis‐acting DNA elements flanking the variable major protein expression site of Borrelia hermsii are required for murine persistence
Source: Microbiologyopen. 2017 Dec 17;7(3):e00569. doi: 10.1002/mbo3.569 (PMC6011951; doi:10.1002/mbo3.569)
Supplement: Supplementary file 6 [file MBO3-7-e00569-s006.pdf]

			1 100
Bh::UHS$_{AS}$ *vmp*$_{Ex}$ Inoc		(1)	TAGGCTAGCGCAAGAGCTATAGCATTAAGAGAATGGCTAAAGGTGATAAATTTGATTTTTTTTTTTTAACTTTGTAACTTTGAAAGTTGAGGTATAAT
Recovered *vmp*$_{Ex}$	SCID	(1)	--AACTTTGTAACTTTGAAAGTTGAGGTATAAT
			101 200
Bh::UHS$_{AS}$ *vmp*$_{Ex}$ Inoc		(101)	TATGACGCAACATTAAGTAGGAGGAAAGCATTTCAATGAGGAAGAGGATCAGCGCGATCATAATGACTTTATTTATGGTATTAGTAAGCTGTAATAGCGG
Recovered *vmp*$_{Ex}$	SCID	(33)	TATGACGCAACATTAAGTAGGAGGAAAGCATTTCAATGAGGAAGAGGATCAGCGCGATCATAATGACTTTATTTATGGTATTAGTAAGCTGTAATAGCGG
			201 300
Bh::UHS$_{AS}$ *vmp*$_{Ex}$ Inoc		(201)	TGGGGTTGCGGAAGACCCTCAGAGTAAATTTTTAAAGTCAGCAATAGACTTAGGTAATGATTTTTTAAATGTGTTTACATCATTTGGAGATATAGTTTCC
Recovered *vmp*$_{Ex}$	SCID	(133)	TGGGGTTGCGGAAGACCCTCAGAGTAAATTTTTAAAGTCAGCAATAGACTTAGGTAATGATTTTTTAAATGTGTTTACATCATTTGGAGATATAGTTTCC
			301 400
Bh::UHS$_{AS}$ *vmp*$_{Ex}$ Inoc		(301)	AAGGTATTAGGTTTTAGTACAGAGACAAAAAAGTCTGATGTTGGGGCTTATTTTAAGACAATACAAGATACTATACAAGGCACTAAGGACAAGCTTAATA
Recovered *vmp*$_{Ex}$	SCID	(233)	AAGGTATTAGGTTTTAGTACAGAGACAAAAAAGTCTGATGTTGGGGCTTATTTTAAGACAATACAAGATACTATACAAGGCACTAAGGACAAGCTTAATA
			401 500
Bh::UHS$_{AS}$ *vmp*$_{Ex}$ Inoc		(401)	AAATTGTTACTGACATGAAGAGAGAAGGAAATCCTAATGCTTCTGCAACTGAGACTGCGGTAAAAACACTAATTGATAATACTCTTGATAAGATAATAGA
Recovered *vmp*$_{Ex}$	SCID	(333)	AAATTGTTACTGACATGAAGAGAGAAGGAAATCCTAATGCTTCTGCAACTGAGACTGCGGTAAAAACACTAATTGATAATACTCTTGATAAGATAATAGA
			501 600
Bh::UHS$_{AS}$ *vmp*$_{Ex}$ Inoc		(501)	AGGTGCTGAGACTGCAAGTGAGGCTATTGGTGATGCTGGTGACCCAATTGGTAATGTTGCTGCTGGTGGTGCTGGTGCGGGTACAGGTGCTATTGGGGAT
Recovered *vmp*$_{Ex}$	SCID	(433)	AGGTGCTGAGACTGCAAGTGAGGCTATTGGTGATGCTGGTGACCCAATTGGTAATGTTGCTGCTGGTGGTGCTGGTGCGGGTACAGGTGCTATTGGGGAT
			601 700
Bh::UHS$_{AS}$ *vmp*$_{Ex}$ Inoc		(601)	GGTGTTGATAATCTAATAAATGGAATTAAGGCAATTGTAGAAGTAGTACTTAAAGAAGGGAATGCTGAGGCTGGAGATGGTAAAAAGGCCGATGCTCTTG
Recovered *vmp*$_{Ex}$	SCID	(533)	GGTGTTGATAATCTAATAAATGGAATTAAGGCAATTGTAGAAGTAGTACTTAAAGAAGGGAATGCTGAGGCTGGAGATGGTAAAAAGGCCGATGCTCTTG
			701 800
Bh::UHS$_{AS}$ *vmp*$_{Ex}$ Inoc		(701)	GAGCAAGAGGTGCTAATGCTGGTGATGCAGGAAAGTTATTTGGTAATACTGGTAATAATGGTGCTATTGATTCTGCAGATAATGCAAAGAAAGCAGGTGC
Recovered *vmp*$_{Ex}$	SCID	(633)	GAGCAAGAGGTGCTAATGCTGGTGATGCAGGAAAGTTATTTGGTAATACTGGTAATAATGGTGCTATTGATTCTGCAGATAATGCAAAGAAAGCAGGTGC
			801 900
Bh::UHS$_{AS}$ *vmp*$_{Ex}$ Inoc		(801)	TGATGCAGCAAAAGCAGTAGGGGCAGTAACAGGTGCTGATATATTACAAGCTATTTCTAAAGATGGTGGTGATGCTGCTAAATTAGCTAAGAATAGTGCT
Recovered *vmp*$_{Ex}$	SCID	(733)	TGATGCAGCAAAAGCAGTAGGGGCAGTAACAGGTGCTGATATATTACAAGCTATTTCTAAAGATGGTGGTGATGCTGCTAAATTAGCTAAGAATAGTGCT
			901 1000
Bh::UHS$_{AS}$ *vmp*$_{Ex}$ Inoc		(901)	ACCGTTCAGGTGACTGGTGTTGCTGTTGATGTTAAAGATGCGGTTATAGCAGGAGGAATTGCACTCAGAGCAATGGCAAAGGGTGGTAAATTTGCTAATG
Recovered *vmp*$_{Ex}$	SCID	(833)	ACCGTTCAGGTGACTGGTGTTGCTGTTGATGTTAAAGATGCGGTTATAGCAGGAGGAATTGCACTCAGAGCAATGGCAAAGGGTGGTAAATTTGCTAATG
			1001 1100
Bh::UHS$_{AS}$ *vmp*$_{Ex}$ Inoc		(1001)	ATAAGGATGCTGTTAATGCTGATGTTGTTACTGCAGTTAAAGGAGCAACAGTAAGTGCAGTAACTAAAGCACTAGATACATTAACTATTGCAATAAGAAA
Recovered *vmp*$_{Ex}$	SCID	(933)	ATAAGGATGCTGTTAATGCTGATGTTGTTACTGCAGTTAAAGGAGCAACAGTAAGTGCAGTAACTAAAGCACTAGATACATTAACTATTGCAATAAGAAA
			1101 1200
Bh::UHS$_{AS}$ *vmp*$_{Ex}$ Inoc		(1101)	AACAATTGACGCAGGCCTTAAAACAGTTAAAGAAGCTATGAAAATTAATGCTAATGATACTCCTATAACTCCTGAGCAGAATATCCCTAAAGCTACTACT
Recovered *vmp*$_{Ex}$	SCID	(1033)	AACAATTGACGCAGGCCTTAAAACAGTTAAAGAAGCTATGAAAATTAATGCTAATGATACTCCTATAACTCCTGAGCAGAATATCCCTAAAGCTACTACT
			1201 1300
Bh::UHS$_{AS}$ *vmp*$_{Ex}$ Inoc		(1201)	AGTAACTAGTTAAGGATAAATATAAAGGATAAAGTCATTGTAAGGGAAAAGCTTTTCTTGTTTTTAATGCAGGAGTGTAGTTTCTCTGATTAAGTAAGCT
Recovered *vmp*$_{Ex}$	SCID	(1133)	AGTAACTAGTTAAGGATAAATATAAAGGATAAAGTCATTGTAAGGGAAAAGCTTTTCTTGTTTTTAATGCAGGAGTGTAGTTTCTCTGATTAAGTAAGCT
			1301 1400
Bh::UHS$_{AS}$ *vmp*$_{Ex}$ Inoc		(1301)	GTAAGAGCAGGGAAAAATAAAGTCAAAAAGGAATAGGAAGCTAGGAGCGTAATGCTCTTAGCTTCTAATGTTATTTAGGGAGTGTTTCTTTGTATATAAA
Recovered *vmp*$_{Ex}$	SCID	(1233)	GTAAGAGCAGGGAAAAATAAAGTCAAAAAGGAATAGGAAGCTAGGAGCGTAATGCTCTTAGCTTCTAATGTTATTTAGGGAGTGTTTCTTTGTATATAAA
			1401 1500
Bh::UHS$_{AS}$ *vmp*$_{Ex}$ Inoc		(1401)	ATTGTTTATATGAGTAAAGATTGAATATAAATAATTGCAAGTATGATATTAAGAGTATGTTTTTATTGTAATCAAATAATTAATACTTTAAAAGTAAGCT
Recovered *vmp*$_{Ex}$	SCID	(1333)	ATTGTTTATATGAGTAAAGATTGAATATAAATAATTGCAAGTATGATATTAAGAGTATGTTTTTATTGTAATCAAATAATTAATACTTTAAAAGTAAGCT
			1501 1576
Bh::UHS$_{AS}$ *vmp*$_{Ex}$ Inoc		(1501)	AAATGTGTGGTAAGGGCAGCAAAAGGGAAATTGGGATAGATGTTGGAAGGAAAAGAAGCACTGGGGATGCGCATAG
Recovered *vmp*$_{Ex}$	SCID	(1433)	AAATGTGTGGTAAGGGCA--
