# Supplementary material for: Cis‐acting DNA elements flanking the variable major protein expression site of Borrelia hermsii are required for murine persistence
Source: Microbiologyopen. 2017 Dec 17;7(3):e00569. doi: 10.1002/mbo3.569 (PMC6011951; doi:10.1002/mbo3.569)
Supplement: Supplementary file 7 [file MBO3-7-e00569-s007.pdf]

```
                                401                                                                                                500
Wild type vmp_Ex Inoc    (401) TTGTTACTGACATGAAGAGAGAAGGAAATCCTAATGCTTCTGCAACTGAGACTGCGGTAAAAACACTAATTGATAATACTCTTG-ATAAGATAATAGAAG
Recovered vmp_Ex SCID      (1) -------------------------------------------AACTTTGTAAACTTTGAAAGT--TGAGGTATAATGCTAATGCATAAGTTAAAAGGAG
                                501                                                                                                600
Wild type vmp_Ex Inoc    (500) GTGCTGAGAC---TGCAAGTGAGGCTATTGGTGATGCTGGTGACCCAATTGGTAATGTTGCTGCTGGTGGTGCTGGTGCGGGTACAGGTGCTATTGGGGA
Recovered vmp_Ex SCID     (56) GCACGTAAAAAAATGAGAAAAAGAATAAGTGCAATAATAATGACTTTATTTATGGTGTTTATG-TCATG-TAATAATGGAGGTCCAGAGCTTAAAAGTGA
                                601                                                                                                700
Wild type vmp_Ex Inoc    (597) TGGTGTTGATAA-TCTAAT--AAATGGAATTAA---GGCAATTGTAGAAGTAGTACTTAAAGAAGGGAATGCTGAGGCTGGAGATGGTAAAAAGGCCGAT
Recovered vmp_Ex SCID    (154) CGAAGTAGCCAAGTCTGACGGAACAGTACTTGATTTGGCAAAAATAAGTGCAAAGATAAAAGAGGCTAGTGCTTTTGCAGCAAGTGTTAAAGAAGTTCAT
                                701                                                                                                800
Wild type vmp_Ex Inoc    (691) GCTCTTGG--AG-CAAGAGGTGCTAATGCTGGTGATGCAGGAAAGTT-ATTTGGTAATACTGGTAATAATGGTGCTATTGATTCTGCAGATAATGCAAAG
Recovered vmp_Ex SCID    (254) ACTTTAGTTAAGTCAGTAGATACGCTTGCTGGTGCTATTGGAAAGAAGATTAAGTCCGA-TGGGAAGTTTGATGCTATGGCTGGTAAGAATGGATCATTG
                                801                                                                                                900
Wild type vmp_Ex Inoc    (787) AAAGCAGGTGCTGATGCAGCAAAAGCAGTAGGGGCAGTAACAGGTGCTGATATATTACAAGCTATTTCTAAAGATGGTGGTGATGCTGCTAAATTAGCTA
Recovered vmp_Ex SCID    (353) CTTGCAGGGGCATAT------AATGTTGCGTTGGATATAAATAGTAAATTGACAGTATTAGATGGT---AAGGCTGG-----ACTCT-CTTCTTTACTTA
                                901                                                                                               1000
Wild type vmp_Ex Inoc    (887) AGAATAGTGCTACCGTTCAGGTGACTGGTGTTGCTGTTGATGTTAAAGATGCGGTTATAGCAGGAGGAATTGCACTCAGAGCAATGGCAAAGGGTGGTAA
Recovered vmp_Ex SCID    (438) AGGCAAAGGTTACTGCTGCAAAAACTAGTGGTGAA--TCATTCTCAA-ATAAATTAAAAACCGAACATACTGACCTTGG--CAAAGA-AGAGGCTAGTGA
                                1001                                                                                              1100
Wild type vmp_Ex Inoc    (987) ATTTGCTAATGATAAGGATGCTGTTAATGCTGATGTTGTTACTGCAGTTAAAGGAGCAACAGTAAGTGCAGTAACTAAAGCACTAGATACATTAACTATT
Recovered vmp_Ex SCID    (532) ---TGATAATGCAAAAGCAGCTTTACTTGTAACAAATGCTACTAAAAATAAAGGGGTCACTGAGCTTG---------AAGCACTCAACACAGCAGTTGAT
                                1101                                                                                              1200
Wild type vmp_Ex Inoc   (1087) GCAATAAGAAAAACAATTGACGCAGGCCTTAAAACAGTTAAAGAAGCTATGAAAATTAATGCTAATGATACTCCTATAACTCCTGAGCAGAATATCCCTA
Recovered vmp_Ex SCID    (620) GCCTTGTTAAAGGCAGCTGAGGGTGA---------AGT---AGAAGCTGC---AATTAAAGAGCTTACAGCTCCTGTTAAG-----GTAGAA-------A
                                1201                                                                                              1300
Wild type vmp_Ex Inoc   (1187) AAGCTACTACTAGTAACTAGTTAAGGATAAATATAAAGGATAAAGTCATTGTAAGGGAAAAGCTTTTCTTGTTTTTAATGCAGGAGTGTAGTTTCTCTGA
Recovered vmp_Ex SCID    (693) AACCTTCTCAAAATAACTAACTAGGGAATAA-ATAATTTAAGAAGTTATTATAAGATAA-----------GTT--------------------------A
                                1301                                                                                              1400
Wild type vmp_Ex Inoc   (1287) TTAAGTAAGCTGTAAGAGCAGGGAAAAATAAAGTCAAAAAGGAATAGGAAGCTAGGAGCGTAATGCTCTTAGCTTCTAATGTTATTTAGGGAGTGTTTCT
Recovered vmp_Ex SCID    (755) TTAAGTAAGCTGTAAGAGCAGGGAAAAATAAAGTCAAAAAGGAATAAGAAGCTAGGAGTGTAATGCTCTTAGCTTCTGATGTTATTTAGGGAATGTTTAT
                                1401                                                                                              1500
Wild type vmp_Ex Inoc   (1387) TTGTATATAAAATTGTTTATATGAGTAAAGATTGAATATAAATAATTGCAAGTATGATATTAAGAGTATGTTTTTATTGTAATCAAATAATTAA-TACTT
Recovered vmp_Ex SCID    (855) TTGTATATAAAATTGTTTATATGAGTAAAGATTGAATATAAATAATTGCAAGTATGATATTAAGAGTATGTTTTTATTGTAATCAAATAATTAAATACTT
                                1501                                                                                1585
Wild type vmp_Ex Inoc   (1486) TAAAAGTAAGCTAAATGTGTGGTAAGGGCAGCAAAAGGGAAATTGGGATAGATGTTGGAAGGAAAAGAAGCACTGGGGATGCGCA
Recovered vmp_Ex SCID    (955) TAAAAGTAAGCTAAATGTGTGGTAAGGGCAGC-----------------------------------------------------
```
