# Supplementary material for: Cis‐acting DNA elements flanking the variable major protein expression site of Borrelia hermsii are required for murine persistence
Source: Microbiologyopen. 2017 Dec 17;7(3):e00569. doi: 10.1002/mbo3.569 (PMC6011951; doi:10.1002/mbo3.569)
Supplement: Supplementary file 8 [file MBO3-7-e00569-s008.pdf]

		1 … 100
Bh::DHS$_{\Delta IR}$ *vmp*$_{Ex}$ Inoc	(1)	TAGGCTAGCGCAAGAGCTATAGCATTAAGAGAATGGCTAAAGGTGATAAATTTGATTTTTTTTTTTTTAACTTTGTAAACTTTGAAAGTTGAGGTATAAT
Recovered *vmp*$_{Ex}$ SCID	(1)	--AACTTTGTAAACTTTGAAAGTTGAGGTATAAT
		101 … 200
Bh::DHS$_{\Delta IR}$ *vmp*$_{Ex}$ Inoc	(101)	GCTAATGCATAAGTTAAAAGGAGGCACGTAAAAAAATGAGAAAAAGAATAAGTGCAATAATAATGACTTTATTTATGGTATTAGTAAGCTGTAATAGCGG
Recovered *vmp*$_{Ex}$ SCID	(33)	GCTAATGCATAAGTTAAAAGGAGGCACGTAAAAAAATGAGAAAAAGAATAAGTGCAATAATAATGACTTTATTTATGGTATTAGTAAGCTGTAATAGCGG
		201 … 300
Bh::DHS$_{\Delta IR}$ *vmp*$_{Ex}$ Inoc	(201)	TGGGGTTGCGGAAGACCCTCAGAGTAAATTTTTAAAGTCAGCAATAGACTTAGGTAATGATTTTTTAAATGTGTTTACATCATTTGGAGATATAGTTTCC
Recovered *vmp*$_{Ex}$ SCID	(133)	TGGGGTTGCGGAAGACCCTCAGAGTAAATTTTTAAAGTCAGCAATAGACTTAGGTAATGATTTTTTAAATGTGTTTACATCATTTGGAGATATAGTTTCC
		301 … 400
Bh::DHS$_{\Delta IR}$ *vmp*$_{Ex}$ Inoc	(301)	AAGGTATTAGGTTTTAGTACAGAGACAAAAAAGTCTGATGTTGGGGCTTATTTTAAGACAATACAAGATACTATACAAGGCACTAAGGACAAGCTTAATA
Recovered *vmp*$_{Ex}$ SCID	(233)	AAGGTATTAGGTTTTAGTACAGAGACAAAAAAGTCTGATGTTGGGGCTTATTTTAAAACAATCCAAGATACTATACAAGGCACTAAGGACAAGCTTAATA
		401 … 500
Bh::DHS$_{\Delta IR}$ *vmp*$_{Ex}$ Inoc	(401)	AAATTGTTACTGACATGAAGAGAGAAGGAAATCCTAATGCTTCTGCAACTGAGACTGCGGTAAAAACACTAATTGATAATACTCTTGATAAGATAATAGA
Recovered *vmp*$_{Ex}$ SCID	(333)	AAATTGTTACTGACATGAAGAGAGAAGGAAATCCTAATGCTTCTGCAACTGAGACTGCGGTAAAAACACTAATTGATAATACTCTTGATAAGATAATAGA
		501 … 600
Bh::DHS$_{\Delta IR}$ *vmp*$_{Ex}$ Inoc	(501)	AGGTGCTGAGACTGCAAGTGAGGCTATTGGTGATGCTGGTGACCCAATTGGTAATGTTGCTGCTGGTGGTGCTGGTGCGGGTACAGGTGCTATTGGGGAT
Recovered *vmp*$_{Ex}$ SCID	(433)	AGGTGCTGAGACTGCAAGTGAGGCTATTGGTGATGCTGGTGACCCAATTGGTAATGTTGCTGCTGGTGGTGCTGGTGCGGGTACAGGTGCTATTGGGGAT
		601 … 700
Bh::DHS$_{\Delta IR}$ *vmp*$_{Ex}$ Inoc	(601)	GGTGTTGATAATCTAATAAATGGAATTAAGGCAATTGTAGAAGTAGTACTTAAAGAAGGGAATGCTGAGGCTGGAGATGGTAAAAAGGCCGATGCTCTTG
Recovered *vmp*$_{Ex}$ SCID	(533)	GGTGTTGATAATCTAATAAATGGAATTAAGGCAATTGTAGAAGTAGTACTTAAAGAAGGGAATGCTGAGGCTGGAGATGGTAAAAAGGCCGATGCTCTTG
		701 … 800
Bh::DHS$_{\Delta IR}$ *vmp*$_{Ex}$ Inoc	(701)	GAGCAAGAGGTGCTAATGCTGGTGATGCAGGAAAGTTATTTGGTAATACTGGTAATAATGGTGCTATTGATTCTGCAGATAATGCAAAGAAAGCAGGTGC
Recovered *vmp*$_{Ex}$ SCID	(633)	GAGCAAGAGGTGCTAATGCTGGTGATGCAGGAAAGTTATTTGGTAATACTGGTAATAATGGTGCTATTGATTCTGCAGATAATGCAAAGAAAGCAGGTGC
		801 … 900
Bh::DHS$_{\Delta IR}$ *vmp*$_{Ex}$ Inoc	(801)	TGATGCAGCAAAAGCAGTAGGGGCAGTAACAGGTGCTGATATATTACAAGCTATTTCTAAAGATGGTGGTGATGCTGCTAAATTAGCTAAGAATAGTGCT
Recovered *vmp*$_{Ex}$ SCID	(733)	TGATGCAGCAAAAGCAGTAGGGGCAGTAACAGGTGCTGATATATTACAAGCTATTTCTAAAGATGGTGGTGATGCTGCTAAATTAGCTAAGAATAGTGCT
		901 … 1000
Bh::DHS$_{\Delta IR}$ *vmp*$_{Ex}$ Inoc	(901)	ACCGTTCAGGTGACTGGTGTTGCTGTTGATGTTAAAGATGCGGTTATAGCAGGAGGAATTGCACTCAGAGCAATGGCAAAGGGTGGTAAATTTGCTAATG
Recovered *vmp*$_{Ex}$ SCID	(833)	ACCGTTCAGGTGACTGGTGTTGCTGTTGATGTTAAAGATGCGGTTATAGCAGGAGGAATTGCACTCAGAGCAATGGCAAAGGGTGGTAAATTTGCTAATG
		1001 … 1100
Bh::DHS$_{\Delta IR}$ *vmp*$_{Ex}$ Inoc	(1001)	ATAAGGATGCTGTTAATGCTGATGTTGTTACTGCAGTTAAAGGAGCAACAGTAAGTGCAGTAACTAAAGCACTAGATACATTAACTATTGCAATAAGAAA
Recovered *vmp*$_{Ex}$ SCID	(933)	ATAAGGATGCTGTTAATGCTGATGTTGTTACTGCAGTTAAAGGAGCAACAGTAAGTGCAGTAACTAAAGCACTAGATACATTAACTATTGCAATAAGAAA
		1101 … 1200
Bh::DHS$_{\Delta IR}$ *vmp*$_{Ex}$ Inoc	(1101)	AACAATTGACGCAGGCCTTAAAACAGTTAAAGAAGCTATGAAAATTAATGCTAATGATACTCCTATAACTCCTGAGCAGAATATCCCTAAAGCTACTACT
Recovered *vmp*$_{Ex}$ SCID	(1033)	AACAATTGACGCAGGCCTTAAAACAGTTAAAGAAGCTATGAAAATTAATGCTAATGATACTCCTATAACTCCTGAGCAGAATATCCCTAAAGCTACTACT
		1201 … 1300
Bh::DHS$_{\Delta IR}$ *vmp*$_{Ex}$ Inoc	(1201)	AGTAACTAGTTAAGGATAAATATAAAGGATAAAGTCATTGTAAGGGAAAAGCTTTTCTTGTTTTTAATGCAGGAGTGTAGTTTCTCTGATTAAGTAAGCT
Recovered *vmp*$_{Ex}$ SCID	(1133)	AGTAACTAGTTAAGGATAAATATAAAGGATAAAGTCATTGTAAGGGAAAAGCTTTTCTTGTTTTTAATGCAGGAGTGTAGTTTCTCTGATTAAGTAAGCT
		1301 … 1400
Bh::DHS$_{\Delta IR}$ *vmp*$_{Ex}$ Inoc	(1301)	GTAAGAGCAGGGAAAAATAAAGTCAAAAAGGAATAAATGTTATTTAGGGAGTGTTTCTTTGTATATAAAATTGTTTATATGAGTAAAGATTGAATATAAA
Recovered *vmp*$_{Ex}$ SCID	(1233)	GTAAGAGCAGGGAAAAATAAAGTCAAAAAGGAATAAATGTTATTTAGGGAGTGTTTCTTTGTATATAAAATTGTTTATATGAGTAAAGATTGAATATAAA
		1401 … 1500
Bh::DHS$_{\Delta IR}$ *vmp*$_{Ex}$ Inoc	(1401)	TAATTGCAAGTATGATATTAAGAGTATGTTTTTATTGTAATCAAATAATTAATACTTTAAAAGTAAGCTAAATGTGTGGTAAGGGCAGCAAAAGGGAAAT
Recovered *vmp*$_{Ex}$ SCID	(1333)	TAATTGCAAGTATGATATTAAGAGTATGTTTTTATTGTAATCAAATAATTAATACTTTAAAAGTAAGCTAAATGTGTGGTAA------------------
		1501 … 1545
Bh::DHS$_{\Delta IR}$ *vmp*$_{Ex}$ Inoc	(1501)	TGGGATAGATGTTGGAAGGAAAAGAAGCACTGGGGATGCGCATAG
Recovered *vmp*$_{Ex}$ SCID	(1415)	---
